# Community Succession and Diversity Variation of Endophytic and Rhizosphere Soil Bacteria Across *Gastrodia elata* Seed Formation Stages

**DOI:** 10.3390/biology15110829

**Published:** 2026-05-25

**Authors:** Kaize Shen, Mingjian Xu, Wei Zhou, Hongyin Zhou, Weihua Wang, Yani Su, Haiyan He, Shunqiang Yang

**Affiliations:** 1 Yunnan Key Laboratory of Gastrodia and Fungi Symbiotic Biology, Zhaotong University, Zhaotong 657000, China; ztushenkz@163.com (K.S.); xumingjian0228@163.com (M.X.); zw18786131893@126.com (W.Z.); zhy1605202632@163.com (H.Z.); 34016@ztu.edu.cn (W.W.); 202415060211@stu.ztu.edu.cn (Y.S.); 2 Yunnan Engineering Research Center of Green Planting and Processing of Gastrodia, Zhaotong University, Zhaotong 657000, China; 3 School of Agronomy and Life Sciences, Zhaotong University, Zhaotong 657000, China; 4 Yunnan Key Laboratory of Smart Villages and Agri-Cultural-Tourism Integration, Zhaotong University, Zhaotong 657000, China

**Keywords:** *Gastrodia elata* Blume, seed formation, endophytic bacteria, rhizosphere soil bacteria, bacterial diversity, Community succession

## Abstract

The *Gastrodia elata* Blume (*GE*) life cycle is unique, since its successful germination and growth rely on symbiosis with specific fungi (e.g., *Armillaria mellea*). However, the community succession, tissue specificity and functional potential of endophytic and rhizosphere bacterial communities during the seed formation stage of *GE* remain unclear. Here, we used high-throughput 16S rRNA gene sequencing to systematically explore the composition, diversity, and dynamic succession of bacterial communities across different stages of seed formation and among various tissues. Our results revealed that the endophytic community remained relatively stable across most developmental stages and tissue types (ANOSIM R = 0.4568, *p* = 0.001), with significant compositional shifts only occurring at the fruiting stage in specific tissues (stems and seeds). In contrast, the rhizosphere soil bacterial community showed stronger developmental succession (ANOSIM R = 0.7037, *p* = 0.001), with progressive divergence and the strongest segregation observed between the initial planting and fruiting stages. Alpha diversity peaked at the flowering stage for endophytic bacteria (Shannon index) and at the bud formation stage for rhizosphere soil bacteria, with persistent core taxa (*Bacteroides* in endophytic bacteria, *Pseudarthrobacter* in rhizosphere soil bacteria) dominating across stages. Functional predictions revealed stable core metabolic pathways, with stage-specific enrichments of glycolysis or gluconeogenesis at late developmental stages. These results provide novel ecological insights into the spatiotemporal dynamics of bacterial communities across different stages of *GE* seed formation, highlighting the distinct ecological strategies of endophytic and rhizosphere soil bacteria during the reproductive development of the plant.

## Simple Summary

*Gastrodia elata* Blume (*GE*) is a valuable medicinal plant that depends on fungi to germinate and grow. However, we know little about the bacteria living inside its tissues and around its roots and their function during the *GE* seed formation. Here, we used DNA sequencing to explore these bacterial communities across five key seed development stages. We found that bacteria living inside the plant remained mostly stable across different stages and tissues, only changing significantly at the fruiting stage in stems and seeds. In contrast, bacteria living in the soil around the roots changed much more dramatically as the *GE* developed, with the strongest difference between the planting and the fruiting stages. Certain bacteria, such as *Bacteroides* inside the *GE* tissues and *Pseudarthrobacter* in the soil, were consistently present throughout. These findings help us better understand how this medicinal plant interacts with microbes and provide useful knowledge for improving the cultivation and conservation of this important medicinal herb.

## 1. Introduction

Orchidaceae plants establish symbiotic associations with microorganisms throughout their life cycle [1], However, while the well-documented function of endophytic fungi in promoting seed germination and vegetative growth is widely recognized, endophytic bacteria remain poorly characterized, particularly during the critical seed formation stage that limits the artificial propagation of many endangered or medicinal Orchidaceae [2,3,4]. Previous studies on orchid-associated bacteria and rhizosphere microbiomes have focused almost exclusively on vegetative tissues and growth stages, examining plant-growth-promoting properties or correlations with host habitat [5,6]. However, these culture-dependent approaches inherently fail to reveal the full diversity of endophytic bacteria [7]. The rise of high-throughput 16S rRNA gene sequencing (HTS) has overcome this limitation, enabling comprehensive profiling of endophytic bacterial communities in medicinal orchids such as *Dendrobium* sp. [8], *Vanilla planifolia* [9] and *Geodorum* sp. [10] and providing more comprehensive community information than culture-based methods. This further supports the application of HTS to systematically characterize bacterial community dynamics during seed formation in *Gastrodia elata* Blume (*GE*).

As a perennial mycoheterotrophic orchid, *GE* is an important medicinal herb in traditional Chinese medicine and is widely distributed in East and South Asia [11,12]. Its life cycle spans approximately three years and is divided into vegetative and reproductive stages. The vegetative stage includes seed germination, protocorm development and mature tuber formation; the reproductive stage encompasses bolting, bud formation, flowering and seed maturation [13]. Notably, *GE* seeds are minute, dust-like and lack nutrient reserves. They require symbiosis with *Mycena* for germination and protocorm formation, while subsequent vegetative growth depends on symbiosis with *Armillaria mellea* [12]. However, after entering the reproductive stage (from arrow-tuber formation to seed maturity), *GE* no longer relies on these two symbiotic fungi [13]. This forms an important yet unexplored research gap concerning the functions of bacterial communities during this crucial reproductive stage.

Based on the sexual reproductive growth cycle of *GE*, seed formation can be clearly classified into five consecutive developmental stages [13], all of which are covered in this study: (i) initial planting stage (arrow tuber placed in soil, not yet sprouted); (ii) seedling emergence stage (sprout appears above ground, tip pointed and slender, no flower buds); (iii) bud formation stage (flower stem apex expands and develops buds after bolting); (iv) flowering stage (from the first blossom opening to the last flower withering); and (v) fruiting stage (initial seed maturation to final seed maturation).

Research on bacterial communities associated with *GE* has progressed slowly in recent years, and most existing studies only cover a limited number of growth stages and ecological niches. One study [14] identified Pseudomonadota, Actinomycetota, and Acidobacteriota as the dominant endophytic bacterial phyla in *GE* tubers collected from different production areas, revealing significant regional variation in community composition. Another study explored dynamic shifts in rhizosphere soil bacterial communities during vegetative growth from seed germination to mature tuber formation and demonstrated clear successional trends in community richness and diversity throughout this growth phase [15]. In addition to studies focusing on bacteria in tubers and the rhizosphere during the vegetative stage, the functional role and diversity of bacteria in the *GE*–*Armillaria* symbiotic system have also been investigated. A recent study [16] by Jin et al. combining high-throughput 16S rRNA sequencing and isolation culture revealed that *Armillaria* rhizomorphs associated with *GE* harbor a highly diverse endophytic bacterial community, with core dominant genera including *Burkholderia-Caballeronia-Paraburkholderia*, *Bradyrhizobium*, and *Yersinia*. The community structure is significantly shaped by both *Armillaria* species identity and soil physicochemical properties (pH, available phosphorus, and available potassium). Functional characterization of 49 isolated strains demonstrated that all produced indole-3-acetic acid (IAA), 14 exhibited phosphate-solubilizing ability and three could hydrolyze potassium, highlighting their plant-growth-promoting potential. Despite these advances, all existing studies on *GE*-associated bacterial communities have focused exclusively on the vegetative growth stage, leaving a critical gap: almost no information is available on the composition, diversity, and functional potential of endophytic and rhizosphere soil bacterial communities during the reproductive stage of seed formation.

Moreover, *GE* is one of the most widely cultivated traditional Chinese medicinal herbs in China, with an annual industrial output value exceeding billions of CNY [17]. Artificial propagation and seed quality improvement represent the core bottlenecks restricting the sustainable development of the *GE* industry, as the low germination rate and unstable quality of artificially cultivated seeds have long plagued both producers and researchers [18]. Crucially, seed formation acts as the pivotal period that determines seed quality. Symbiotic associations between *GE* and its associated microorganisms during this period serve as core regulators of seed development and quality formation. Therefore, exploring the bacterial community dynamics during the seed formation of *GE* is not only of great theoretical significance but also an urgent practical need to break the core bottlenecks restricting its cultivation industry.

To bridge this research gap, we systematically investigated the bacterial communities associated with *GE* across its entire seed formation process. As the first research to comprehensively reveal the dynamic succession of both endophytic and rhizosphere soil bacterial communities throughout the complete reproductive seed formation cycle of *GE* using high-throughput 16S rRNA gene sequencing, we aimed to: (i) clarify the composition, diversity, and successional dynamics of these communities across the five stages; (ii) to compare community structure across developmental stages and tissue compartments, identifying key stage- and tissue-specific bacterial taxa; (iii) to predict the functional capacity of principal bacterial communities and explore their putative significance in regulating growth, development and seed maturation during the reproductive stage. We further hypothesized that (i) both endophytic and rhizosphere bacterial communities would present significant stage- and tissue-specific successional dynamics; (ii) the core taxa enriched at different stages would be closely linked to the physiological and nutritional requirements of *GE* during seed formation; (iii) these bacterial communities would play critical functional roles in regulating reproductive development and seed maturation, especially during the late stages when *GE* no longer relies on traditional symbiotic fungi. Filling this knowledge gap is of both theoretical and practical importance: it will advance our understanding of plant–microbe symbiotic mechanisms during reproductive development in mycoheterotrophic orchids and also targeting seed formation—the core bottleneck for artificial propagation, germplasm improvement and sustainable cultivation of the widely cultivated medicinal plant *GE*.

## 2. Materials and Methods

### 2.1. Design of the Experiment, Collection of Samples, and Plant Growth Environment

#### 2.1.1. Study Site and Initial Sample Collection

Initial tubers of *Gastrodia elata* Blume (*GE*) and their associated planting soil were collected in March 2024 from a commercial planting base (Shanjiang Planting Professional Cooperative, Zhaotong, China) in Lianfeng Town, Yongshan County, Zhaotong City, China (103°39′–103°40′ E, 27°53′–27°54′ N). Standard S-shaped sampling was adopted to guarantee sample representativeness. Healthy tubers were selected for subsequent cultivation according to the following criteria: plump terminal buds with bright red color, no visible insect damage, disease spots, or mechanical injury, and fresh weight ranging from 0.12 to 0.13 kg. Selected tubers were immediately transported to the laboratory in an ice box for planting.

#### 2.1.2. Experimental Design

A pot cultivation experiment was conducted using a completely randomized design to obtain *GE* samples at four consecutive seed developmental stages. Each plastic breeding pot (Taizhou Longji Plastic Co., Ltd., Taizhou, China) (0.42 m × 0.27 m × 0.21 m, with bottom drainage holes) served as one independent experimental unit. Eleven healthy tubers were planted in each pot following unified planting specifications. Four independent replicate pots were established for each of the four developmental stages, totaling 16 pots. At each stage, three healthy plants were randomly selected from each replicate pot, and different tissue types (e.g., epidermis, internal tissue, stem, floral stalk, flower and seed) were separated from each plant. For each tissue type per stage, three independent DNA samples (one from each biological replicate pot) were used to construct independent 16S rRNA gene sequencing libraries [19]; no sample pooling was performed, ensuring the independence of biological replicates. A comprehensive sample coding system was adopted (Table S1).

#### 2.1.3. Standardized Cultivation Conditions

**Figure 1:**
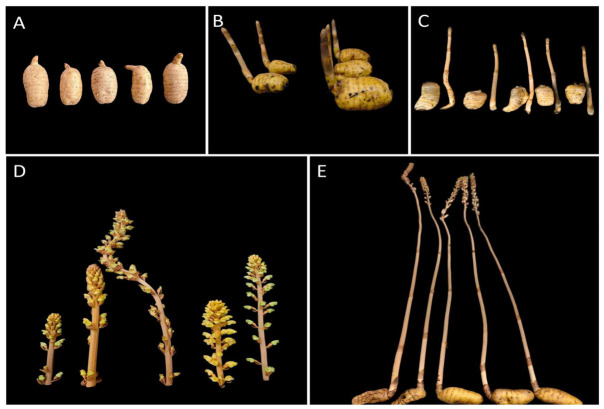
Morphological characteristics of *GE* at five seed developmental stages: (**A**) initial planting (GS1), (**B**) seedling emergence (GS2), (**C**) bud formation (GS3), (**D**) flowering (GS4), and (**E**) fruiting (GS5). All samples were collected from the same planting base under consistent soil, climate, and field management conditions.

All pots were placed in a cool and well-ventilated indoor culture room, where stable and standardized environmental conditions were maintained during the whole experiment. The cultivation substrate was the field-collected planting soil. Daytime ambient temperatures were kept between 18 and 22 °C, whereas nighttime values were maintained at 12–15 °C, providing a diurnal temperature difference of 6–10 °C that mimics natural growth conditions. Relative humidity was kept at 70–80%. Sterile deionized water was used for irrigation. The watering frequency was adjusted to once or twice weekly according to real-time soil moisture monitoring to prevent waterlogging and drought stress. The five developmental stages were defined based on morphological characteristics of *GE* and were sampled at the following standardized time intervals after planting: initial planting stage (GS1) at 0 days after planting (DAP); seedling emergence stage (GS2) at 45 DAP, when aerial stems emerged; bud formation stage (GS3) at 52 DAP, when inflorescence buds fully developed; flowering stage (GS4) at 60 DAP, at full bloom; and fruiting stage (GS5) at 85 DAP, when seeds and tubers fully matured (Figure 1).

### 2.2. Sample Processing

#### 2.2.1. Plant Tissue Processing

Tissues of *GE* collected at five successive developmental stages were dissected into above-ground and under-ground parts with sterile scissors (Suzhou Shilai Medical Devices Co., Ltd., Suzhou, China). Surface-attached soil was rinsed off with sterile deionized water, followed by standardized surface sterilization of all tissue samples. Briefly, the tissues were first immersed in 75% (*v*/*v*) ethanol (Sinopharm Chemical Reagent Co., Ltd., Shanghai, China) for 2 min to eliminate surface epiphytic microorganisms, followed by immersion in a 5% (*w*/*v*) sodium hypochlorite (NaClO) solution (Sinopharm Chemical Reagent Co., Ltd., Shanghai, China) for 5 min for deep surface disinfection. A secondary immersion in 75% (*v*/*v*) ethanol for 30 s was then performed to remove residual NaClO, and the tissues were finally rinsed three consecutive times with sterile deionized water to completely eliminate residual disinfectants [20,21].

The efficacy of surface sterilization was verified using the standard plate-coating method [22]: 100 μL of the final sterile water rinsing solution was evenly spread on Luria–Bertani (LB) solid medium (Hopebio Technology Co., Ltd., Qingdao, China), and the plates were incubated at 37 °C for 48 h. Absence of bacterial colonies on the culture plates indicated successful surface sterilization, which confirmed the thorough elimination of surface epiphytic microbes.

After surface sterilization, 3 g of each tissue sample was accurately weighed and placed into sterile 50 mL centrifuge tubes. All samples were kept at −80 °C in an ultra-low-temperature freezer (Thermo Fisher Scientific, Waltham, MA, USA) until subsequent DNA extraction and analysis.

#### 2.2.2. Rhizosphere Soil Sample Processing

Rhizosphere soil samples were collected following the standard protocol for plant rhizosphere microbiome research [23]. Loose bulk soil on tuber surfaces was gently shaken off, and tightly bound rhizosphere soil was carefully collected with a sterile brush. Subsequently, 3 g of rhizosphere soil from each sample was weighed and placed into sterile 50 mL centrifuge tubes then stored at −80 °C in an ultra-low-temperature refrigerator (Thermo Fisher Scientific, Waltham, MA, USA) for subsequent DNA extraction and analyses.

### 2.3. DNA Extraction, PCR Amplification and High-Throughput Sequencing

We prepared total DNA by extracting it from surface-sterilized *GE* seed tissues and from rhizosphere soil samples collected across different developmental stages using an E.Z.N.A.^®^ Plant DNA Kit and the E.Z.N.A.^®^ Soil DNA Kit (Omega Bio-tek, Norcross, GA, USA), respectively. Extraction blank controls consisting of sterile deionized water without any tissue or soil samples were set up in each batch to rule out exogenous contamination during DNA extraction.

We used the universal primer pair 799F (5′-AACMGGATTAGATACCCKG-3′) and 1193R (5′-ACGTCATCCCCACCTTCC-3′) (Thermo Fisher Scientific, Waltham, MA, USA) to amplify the V3–V4 hypervariable region of the bacterial 16S rRNA gene [24]. Each primer was attached to a distinct 8 bp barcode to facilitate sample multiplexing, and sequencing PCR amplification was carried out in a 20 μL reaction system, which contained 4 μL of 5× TransStart FastPfu Buffer (TransGen Biotech, Beijing, China), 2 μL of 2.5 mM dNTPs (TransGen Biotech, Beijing, China), 0.8 μL of each primer (5 μM), 0.4 μL of TransStart FastPfu DNA Polymerase (TransGen Biotech, Beijing, China), 10 ng of template DNA, and nuclease-free ddH_2_O to adjust the final volume to 20 μL. The PCR thermal cycling conditions were set as follows [25]: initial denaturation at 95 °C for 3 min followed by 27 cycles of 95 °C for 30 s, annealing at 55 °C for 30 s, and extension at 72 °C for 30 s, with a final extension at 72 °C for 10 min. Amplified products were held at 4 °C pending further processing. To control for PCR-related contamination, PCR negative controls (nuclease-free ddH_2_O instead of sample DNA) were included in each batch of reactions. No visible amplicons were detected in any of the PCR-negative controls by 2% agarose (Thermo Fisher Scientific, Waltham, MA, USA) gel electrophoresis, confirming the absence of contamination during PCR amplification.

High-throughput paired-end sequencing was performed on the Illumina NextSeq 2000 platform (Illumina, San Diego, CA, USA) following the standard operating protocol of (Majorbio Bio-Pharm Technology Co., Ltd., Shanghai, China). The raw sequencing reads obtained from this study were submitted to the National Center for Biotechnology Information (NCBI) Sequence Read Archive (SRA) under BioProject accession PRJNA1440932. Sample-specific SRA accessions ranged from SRR37717734 to SRR37717802 (accessed 21 May 2026).

### 2.4. Processing and Analysis of Data

#### 2.4.1. 16S rRNA Gene Sequencing Data Processing

Using a standardized bioinformatics pipeline implemented in QIIME2 v2023.5 (https://qiime2.org), we processed the raw sequencing data. Briefly, primer and adapter sequences were trimmed by Cutadapt v4.7 (https://cutadapt.readthedocs.io/) with a maximum primer mismatch of 10% [26,27]. Quality control and filtering were then performed with fastp v0.19.6 under the following criteria: reads with an average Phred quality score below 20, reads shorter than 100 bp, or reads containing ambiguous bases (N) were discarded; additionally, the first and last 10 bp of each read were trimmed to remove low-quality terminal bases [28]. After filtering, we used FLASH v1.2.11 to merge paired-end reads. The minimum overlap length was set to 10 bp, and the maximum mismatch ratio within overlapping regions was limited to 0.2 [29]. The merged reads were then screened for chimeras using the UCHIME v4.2 algorithm implemented in UPARSE v7.1, with the SILVA 138 16S rRNA gene reference database (https://www.arb-silva.de/) used for chimera detection and removal [30].

Non-chimeric, high-quality sequences were clustered into operational taxonomic units (OTUs) at a 97% sequence similarity threshold using UPARSE v7.1. This threshold is the internationally accepted standard for prokaryotic species classification based on 16S rRNA gene sequences, ensuring effective discrimination of distinct bacterial species and consistency with the analytical norms of most microbiome studies [31]. Sequences of non-bacterial origin (including chloroplasts, mitochondria, and archaea) were subsequently excluded. Representative sequences from each OTU were taxonomically classified using the RDP classifier v2.11 with a confidence threshold of 0.7 against the SILVA 138 database [32].

To address differences in sequencing depth among samples, the OTU table was rarefied (normalized) based on the minimum valid sequence count per sample: 57,853 sequences for endophytic bacterial samples and 67,115 sequences for rhizosphere soil bacterial samples. The rarefied OTU table was used for all downstream analyses of diversity and community composition, and all data are presented as mean values of biological replicates.

#### 2.4.2. Statistical Analysis

All statistical analyses were carried out with R v4.5.2 (https://www.r-project.org/), mothur v1.48.0 (https://mothur.org/), and vegan v2.6-4 (R package). Significance was defined as a *p*-value less than 0.05, while *p* < 0.01 was considered highly significant. For alpha diversity analysis, the Shannon, Simpson, Chao1, and ACE indices were calculated using mothur v1.30.2 to evaluate bacterial community richness and evenness within each sample [33]. Normality of the index data was first assessed using the Shapiro–Wilk test [34]. For non-normally distributed data, the Kruskal–Wallis H test was used for multi-group comparisons, and Dunn’s test was adopted for subsequent pairwise post hoc pairwise multiple comparisons to assess significant differences in alpha diversity indices among samples from different developmental stages and tissue compartments. The vegan v2.6-4 package was used to perform beta diversity analysis [35]. A Bray–Curtis distance matrix was generated to quantify dissimilarities in bacterial community structure between samples. Principal coordinate analysis (PCoA) was then used for dimensionality reduction to visualize similarities in community composition across samples [19]. To test the significance of groupwise differences in community structure, ANOSIM (analysis of similarities) and PERMANOVA (permutational multivariate analysis of variance) were used, each with 999 permutations [36]. Linear discriminant analysis effect size (LEfSe) was employed to screen differentially enriched bacterial biomarkers in specific developmental stages and tissue compartments. The screening criteria were an LDA score ≥ 2.5 and *p* < 0.05 [37]. Functional prediction of the bacterial communities was performed using Tax4Fun2 v0.3.1 based on the 16S rRNA gene sequences. Predicted metabolic pathways were annotated against the Kyoto Encyclopedia of Genes and Genomes (KEGG) database, and the relative abundances of KEGG level-3 metabolic pathways were extracted for subsequent comparative analysis [38].

#### 2.4.3. Data Visualization Methods

All data visualization was carried out using R v4.5.2 together with its associated specialized packages. Rarefaction curves, species accumulation curves, and UpSet diagrams were generated using ggplot2 v3.4.4 (R package), VennDiagram v1.7.3 (R package), and UpSetR v1.4.0 (R package), respectively, to assess sequencing depth sufficiency and to characterize OTU distribution patterns across samples [39]. Community composition bar charts and heatmaps were produced with the ggplot2 and pheatmap packages, illustrating the compositional variation and relative abundance of bacterial communities across developmental stages and tissue compartments. Principal coordinate analysis (PCoA) ordination plots were drawn using the ggplot2 and vegan packages to visualize sample similarities in bacterial community structure. The same ggplot2 package was used to generate two key charts for core community identification. Finally, LEfSe LDA score bar plots were created with the built-in visualization tool of the LEfSe v1.1.0 and subsequently refined using the ggplot2 package in R [40].

## 3. Results

### 3.1. 16S rRNA Gene Sequencing Data Summary

Following the removal of low-quality and repetitive sequences, a total of 5,368,589 high-quality sequences were generated from all samples, with an average sequence length of 375 bp for bacterial amplicons. Of these total sequences, 4,240,321 belonged to endophytic bacterial samples, averaging 375 bp in length, with the remaining 1,128,268 sequences being derived from rhizosphere soil bacterial samples, with an average length of 377 bp. Subsequently, the relative abundance of operational taxonomic units (OTUs) was standardized based on the minimum sequence count across all samples, which was 57,853 for endophytic bacterial samples and 67,115 for rhizosphere soil bacterial samples. OTU clustering was performed using a sequence similarity threshold of 97%, and after the removal of chloroplast- and mitochondria-derived sequences, a total of 1643 non-redundant OTUs were identified across all samples (Table S2). Of these, 1151 OTUs were specific to the endophytic bacterial community (Table S3), and 492 OTUs were unique to the rhizosphere soil bacterial community (Table S4). Furthermore, rarefaction curves were constructed to evaluate the sufficiency of sequencing depth, and the results revealed a consistent saturation pattern across all samples. Species richness increased rapidly with the number of sequencing reads up to 5000 reads, after which the growth rate gradually slowed. The curves reached a plateau at approximately 15,000 to 20,000 reads, indicating that species accumulation stabilized despite further increases in sequencing depth. Finally, these results confirmed that the volume of sequencing data generated in this study adequately captured the majority of bacterial species present in the samples and reliably reflected the actual community composition in all samples. Thus, the sequencing depth employed in this study is sufficient for subsequent comparative analyses of microbiota structure and diversity (Figure S1).

### 3.2. α-Diversity of Bacterial Communities Across Developmental Stages and Tissues

Overall, the five seed developmental stages exerted distinct effects on the α-diversity of seed endophytic and rhizosphere soil bacterial communities. For endophytic bacteria, community diversity (Shannon index) peaked and evenness (Simpson index) reached its minimum at the flowering stage (GS4), representing a significant increase in diversity compared to the fruiting stage (GS5; *p* < 0.05). Community richness (Ace and Chao indices) also reached its maximum at GS4, though no significant differences were detected across other developmental stages (*p* > 0.05). In contrast, rhizosphere soil bacteria exhibited the highest diversity and richness at the bud formation stage (GS3), with a significant difference in richness observed between GS3 and the fruiting stage (GS5; *p* < 0.05), while no significant differences were found relative to other stages (Table 1).

Across growth stages, the α-diversity of endophytic bacterial communities displayed tissue specificity, with no significant differences detected among most tissues during the majority of developmental periods (*p* > 0.05). The most prominent tissue-specific exception occurred at the fruiting stage (GS5): the epidermis (P0) exhibited not only the highest diversity and richness among all tissues but also significantly greater diversity than the stem (S4) and seed (F4) (*p* < 0.05), with no significant difference in richness observed across tissues at this stage (Table S5).

In detail, dynamic changes in α-diversity across stages were observed for individual tissues, with only statistically significant contrasts highlighted here. For the epidermis, diversity peaked at the fruiting stage (GS5), significantly exceeding that at the initial planting (GS1), seed emergence (GS2), and flowering (GS4) stages (*p* < 0.05); richness reached its highest values at the flowering stage (GS4, Ace index) and fruiting stage (GS5, Chao index), with a significant difference detected between GS4 and GS1 (*p* < 0.05) (Table S6). For internal tissues, diversity also peaked at GS5, showing a significant difference from GS1 (*p* < 0.05), while richness was highest at GS2 (Ace index) and GS5 (Chao index) (Table S7). Stem tissue diversity was greatest during the flowering period (GS4), significantly exceeding that at the fruiting stage (GS5; *p* < 0.05), with richness peaking at GS3 (Ace index) and GS2 (Chao index) (Table S8). Among reproductive tissues (floral stalks, flowers, and seeds), flowers exhibited the highest diversity and richness, significantly surpassing seeds (*p* < 0.05), with richness also differing significantly from floral stalks (*p* < 0.05) (Table S9).

### 3.3. Taxonomic Composition and Successional Dynamics of Bacterial Communities

#### 3.3.1. Composition and Successional Dynamics of Endophytic Bacterial Communities at Different Stages

**Figure 2:**
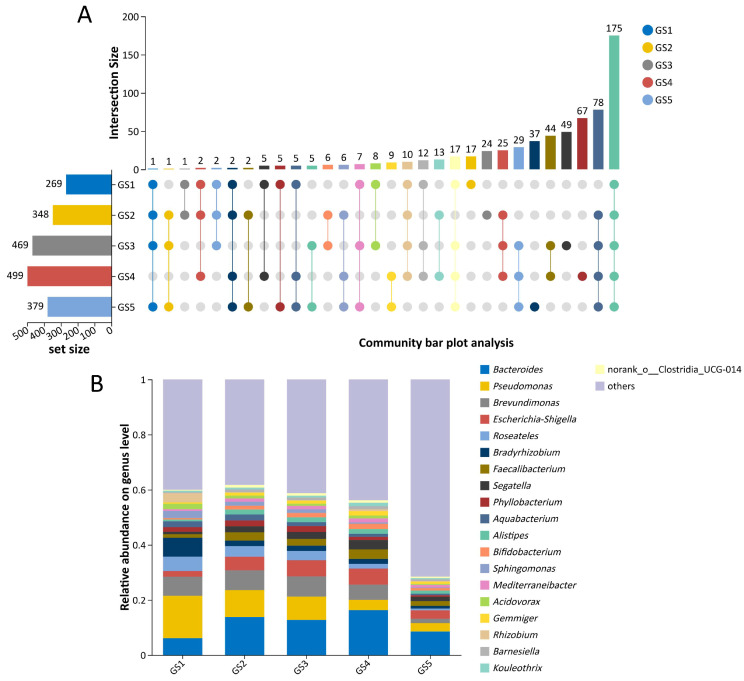
Spatiotemporal distribution of OTUs and genus-level taxonomic composition of endophytic bacterial communities across five seed developmental stages (GS1–GS5) of *GE*. (**A**) UpSet plot showing shared and stage-specific OTUs: left horizontal bars indicate total OTUs per stage (set size); upper vertical bars show intersection sizes; bottom dot matrix indicates which stages are included in each intersection. (**B**) Stacked bar plot of dominant bacterial genus relative abundance; genera with mean relative abundance < 1% across all samples are pooled into “others”. GS1: initial planting; GS2: seedling emergence; GS3: bud formation; GS4: flowering; GS5: fruiting.

The total number of detected OTUs exhibited an overall upward trend from the initial planting stage (GS1) to the flowering stage (GS4), reaching a peak at the flowering stage, followed by a slight decline at the fruiting stage (GS5). This result reflected a gradual increase in the structural complexity of the endophytic bacterial community during seed development, followed by a simplification at the fruiting stage (Figure 2A).

Across all developmental stages, no significant phylum-level replacement was detected across the seed development process, indicating a stable core taxonomic structure of the endophytic community. We identified a total of 546 bacterial genera across all samples, which were taxonomically classified into 23 phyla, 50 classes, 121 orders, and 235 families. Of these, 428 genera belonging to 19 phyla, 39 classes, 98 orders, and 185 families were identified as endophytic bacteria. At the phylum level, Pseudomonadota, Bacteroidota, and Bacillota were the dominant bacterial groups across all developmental stages, collectively accounting for 86.99–95.45% of the total bacterial relative abundance in each stage. The relative abundance of these dominant phyla showed clear stage-specific dynamic changes: Pseudomonadota was the most dominant phylum at the initial planting and fruiting stages, while Bacteroidota and Bacillota exhibited increasing relative abundance from the initial planting stage to the flowering stage, becoming the dominant components during the vegetative growth and reproductive transition phases (Figures S2 and S3).

At the genus level, the bacterial community showed significant successional dynamics across the seed formation cycle. The total number of bacterial genera increased continuously from the initial planting stage (GS1, 155 genera) to the flowering stage (GS4, 237 genera), reflecting a gradual increase in the complexity of the bacterial community structure; the number of genera slightly decreased to 196 at the fruiting stage (GS5). *Bacteroides* was the only dominant genus that maintained high relative abundance throughout all developmental stages, serving as the core persistent genus of the bacterial community during seed formation. Other dominant genera showed clear stage-specific enrichment patterns: *Pseudomonas*, *Brevundimonas*, and *Bradyrhizobium* were the dominant genera at the initial planting stage; *Escherichia-Shigella* became increasingly enriched from the seedling emergence stage (GS2) to the flowering stage (GS4) and was one of the core dominant genera during the reproductive growth phase (Figure 2B and Figure S4).

**Figure 3:**
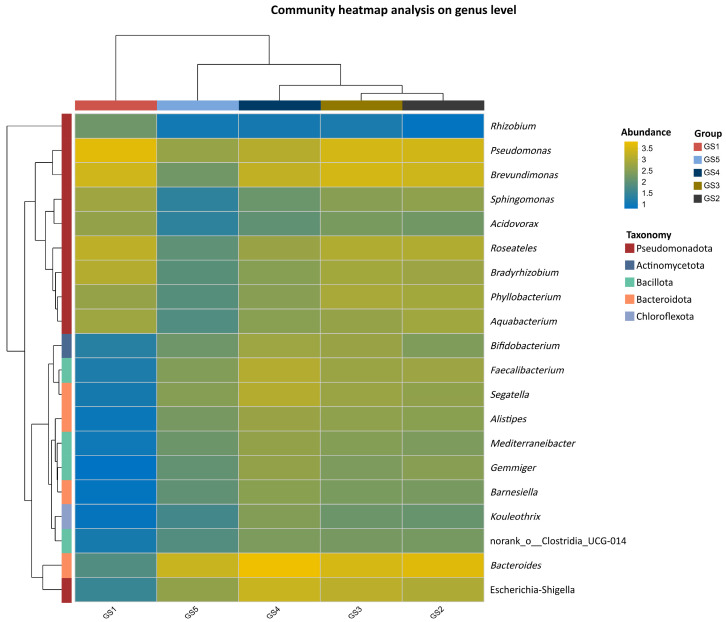
Hierarchical clustering and heatmap of endophytic bacterial communities (genus level) across five seed developmental stages (GS1–GS5) of *GE*. Rows represent genera; columns represent stages. Color gradient (yellow, high abundance; blue, low abundance) indicates relative abundance. Hierarchical clustering trees above (stages) and left (genera) reflect sample community similarity and genus abundance correlation, respectively. Top and left color bars denote developmental stage and phylum-level affiliation of each genus. GS1: initial planting; GS2: seedling emergence; GS3: bud formation; GS4: flowering; GS5: fruiting.

Cluster heatmap analysis at the genus level revealed distinct stage-specific shifts in the endophytic bacterial community of *Gastrodia elata* Blume (*GE*). The community structure at the initial planting stage (GS1) formed a separate branch, markedly different from all later stages. In contrast, structures from seedling emergence to fruiting (GS2–GS5) clustered together, with the highest similarity observed between GS2 and GS3. In terms of species abundance, *Pseudomonas*, *Brevundimonas*, and *Roseateles* remained relatively abundant across multiple stages. *Bacteroides* abundance increased from GS2 onward and became dominant at flowering (GS4). *Escherichia*-*Shigella* showed relatively high abundance during both bud formation (GS3) and flowering (GS4). These results indicate that the endophytic bacterial community exhibits strong stage specificity, with a unique structure at the initial planting stage followed by continuity and similarity among subsequent stages (Figure 3).

#### 3.3.2. Composition and Successional Dynamics of Endophytic Bacterial Communities in Different Tissues

**Figure 4:**
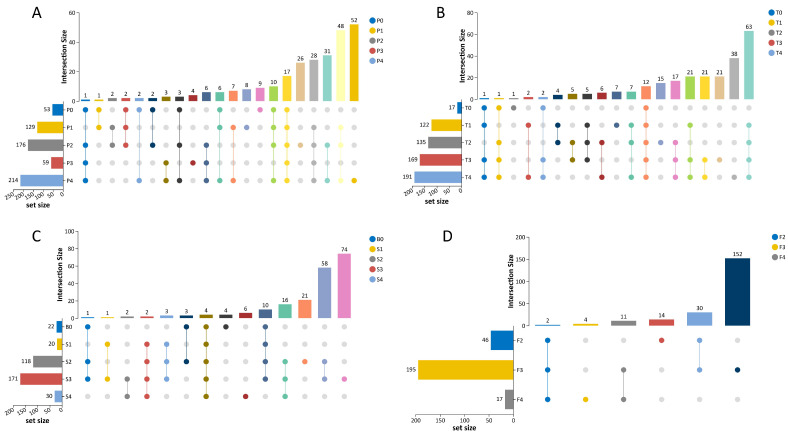
UpSet plots showing intersection patterns of bacterial OTUs across different tissue compartments and developmental stages of *GE* seeds. (**A**) Epidermis (P0–P4); (**B**) internal tissue (T0–T4); (**C**) stem (B0–S4); (**D**) reproductive tissues: floral stalk, flower, and seed (F2–F4). The left horizontal bars indicate total OTU count (set size) per group; the upper vertical bars show intersection sizes (number of shared OTUs). The dot matrix below each vertical bar indicates which sample groups are included in that intersection (solid dot = included, empty dot = not included). Tissue codes P0–P4, T0–T4, and B0–S4 correspond to the five developmental stages (initial planting to fruiting). Codes F2–F4 correspond to the bud formation, flowering, and fruiting stages, respectively.

Overall, all tissue types harbored a stable core bacterial microbiome, with core OTU numbers varying significantly across compartments and stages. Epidermal OTU richness ranged from 53 to 214 across five stages, with 17 shared OTUs. Internal tissue richness increased from 17 to 191, with 12 conserved OTUs. Stem tissues at flowering exhibited the highest core OTU count among stem samples, and reproductive tissues at flowering showed the largest core microbiome overall (152 shared OTUs) (Figure 4).

The endophytic bacterial community was consistently dominated at the phylum level by Pseudomonadota, Bacteroidota, and Bacillota, together accounting for >90% of total relative abundance throughout seed formation. Pseudomonadota was the most dominant phylum overall, with particularly high abundance in the initial planting epidermis (P0, 88.57%), fruiting-stage stem (S4, 94.13%), and fruiting-stage flower (F4, 93.53%). Bacteroidota and Bacillota were predominant in all other tissues and stages, with their abundances fluctuating dynamically (Figures S5 and S6).

**Figure 5:**
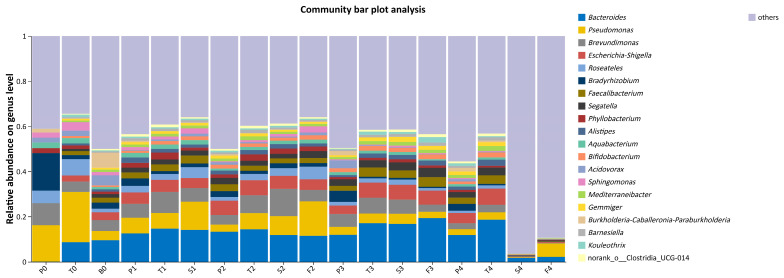
Stacked bar plot depicting how abundant endophytic bacteria relative to one another at the genus level vary among different tissues across five seed developmental stages (GS1–GS5) of *GE*. Each color represents a genus; genera with mean relative abundance < 1% across all samples are pooled into “others”. Tissue codes: epidermis (P0–P4), internal tissue (T0–T4), and stem (B0–S4) correspond to GS1–GS5, respectively; reproductive tissues (F2–F4: floral stalk, flower, seed) correspond to GS3–GS5, respectively.

At the genus level, community composition and relative abundance exhibited clear spatiotemporal dynamics. *Bacteroides* was the most universally dominant and stable genus across all stages, serving as the core endophytic genus, albeit with tissue- and stage-specific variations. During early vegetative stages (initial planting and seedling emergence), the community was co-dominated by *Bradyrhizobium*, *Pseudomonas*, *Brevundimonas*, and *Escherichia*-*Shigella*, with notable tissue-specific differences. Upon entering reproductive stages (bud formation, flowering, and fruiting), *Bacteroides* gradually became the absolute dominant genus across most tissues, except in the fruiting-stage stem (S4) and seed (F4), where its relative abundance markedly decreased (Figure 5 and Figure S7).

**Figure 6:**
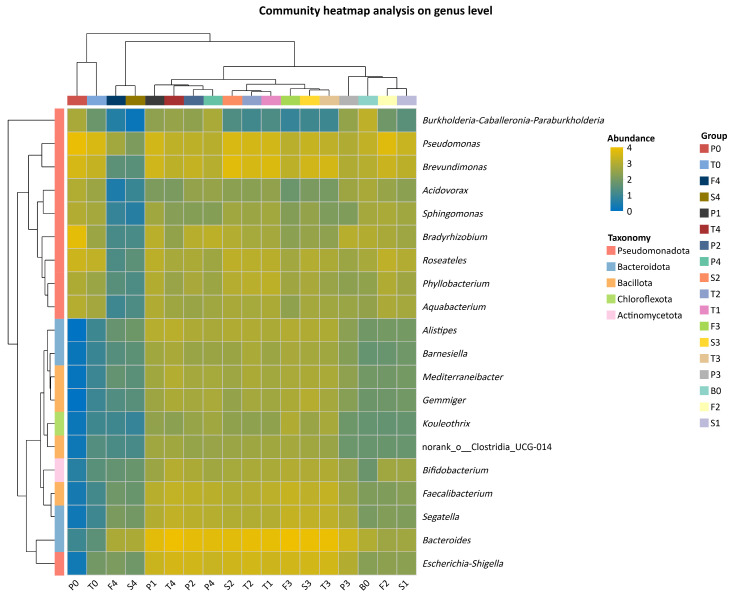
Heatmap depicting hierarchical clustering of endophytic bacterial communities (genus level) across five temporal seed stages (GS1–GS5) of *GE.* Rows represent genera; columns represent tissue samples from different stages (epidermis P0–P4, internal tissue T0–T4, stem B0–S4, and reproductive tissues F2–F4: floral stalk, flower, seed). Color gradient (yellow, high abundance; blue, low abundance) indicates relative abundance. Hierarchical clustering trees above (samples) and left (genera) reflect sample community similarity and genus abundance correlation, respectively. Top and left color bars denote developmental stage and phylum-level affiliation of each genus.

Horizontal cluster analysis at the genus level revealed that endophytic bacterial communities across all tissue samples formed three primary clusters, closely associated with both developmental stage and tissue type. At the initial planting stage, samples from different tissues (P0, T0, and B0) did not cluster closely, with P0 showing the greatest distance due to high *Bradyrhizobium* abundance. Samples at the seedling emergence stage (P1, T1, and S1) clustered relatively tightly, driven by core genera such as *Bacteroides* and *Pseudomonas*. During the bud formation (P2, T2, S2, and F2) and flowering (P3, T3, S3, and F3) stages, samples clustered more closely together, indicating similar community structures across tissues within these reproductive stages; *Bacteroides* and *Escherichia*-*Shigella* formed distinct clusters at budding, while flowering samples consolidated into a single branch. At the fruiting stage, F4 and S4 formed independent, tightly clustered branches, whereas P4 and T4 were characterized predominantly by *Bacteroides*. The clustering distance between F4 and S4 was relatively large, resulting in two major clusters during this stage (Figure 6).

#### 3.3.3. Composition of and Successional Dynamics Rhizosphere Soil Communities at Different Periods

**Figure 7:**
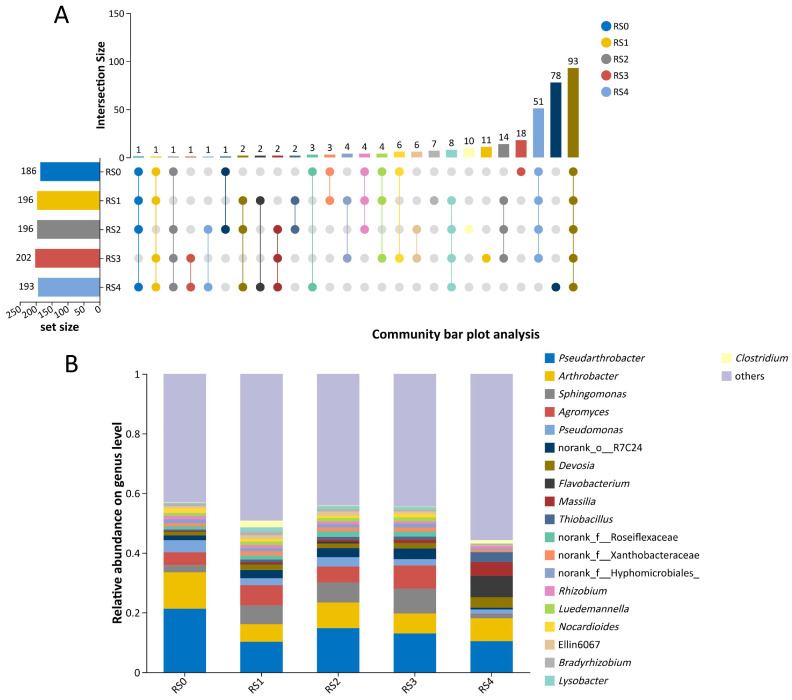
Spatiotemporal distribution of OTUs and genus-level taxonomic composition of rhizosphere soil bacterial communities across five seed developmental stages of *GE* (RS0–RS4: initial planting, seedling emergence, bud formation, flowering, and fruiting). (**A**) UpSet plot showing shared and stage-specific OTUs: left horizontal bars indicate total OTUs per stage (set size); upper vertical bars show intersection sizes; bottom dot matrix indicates which stages are included in each intersection. The colors of the dots and bars correspond to the five stages (RS0: blue; RS1: yellow; RS2: gray; RS3: red; RS4: light blue). (**B**) Stacked bar plot of dominant bacterial genus relative abundance; genera with mean relative abundance < 1% across all samples are pooled into “others”.

The rhizosphere soil bacterial community of *GE* maintained a stable core set of persistent OTUs across all seed developmental stages, alongside significant stage-specific compositional shifts. All five stages together shared 93 OTUs, representing the stable core microbiota (Figure 7A).

Community diversity increased gradually during seed formation. At the phylum level, Pseudomonadota and Actinomycetota consistently dominated (70–90% of relative abundance), with Bacteroidota and Bacillota as subdominant phyla. At the fruiting stage, Pseudomonadota reached 51.79% abundance, followed by Actinomycetota (22.09%), Bacteroidota (12.57%), and Bacillota (6.58%). During the other four stages, Pseudomonadota accounted for 40.92–52.18% and Actinomycetota for 32.67–48.18% (Figures S8 and S9).

At the genus level, total detected genera increased from 111 (initial planting) to 126 (fruiting), reflecting rising diversity. *Pseudarthrobacter* was the most universally dominant genus across all stages. *Sphingomonas*, *Arthrobacter*, and *Agromyces* were consistently present as subdominant taxa. During early stages (initial planting to bud formation), the community was co-dominated by *Pseudarthrobacter*, *Arthrobacter*, and *Agromyces*, with *Sphingomonas* gradually increasing. At flowering and fruiting, *Pseudarthrobacter* remained dominant, but *Sphingomonas* became the second most abundant at flowering, and new genera (*Flavobacterium*, *Massilia*, and *Devosiella*) emerged at fruiting, driving major compositional changes (Figure 7B and Figure S10).

**Figure 8:**
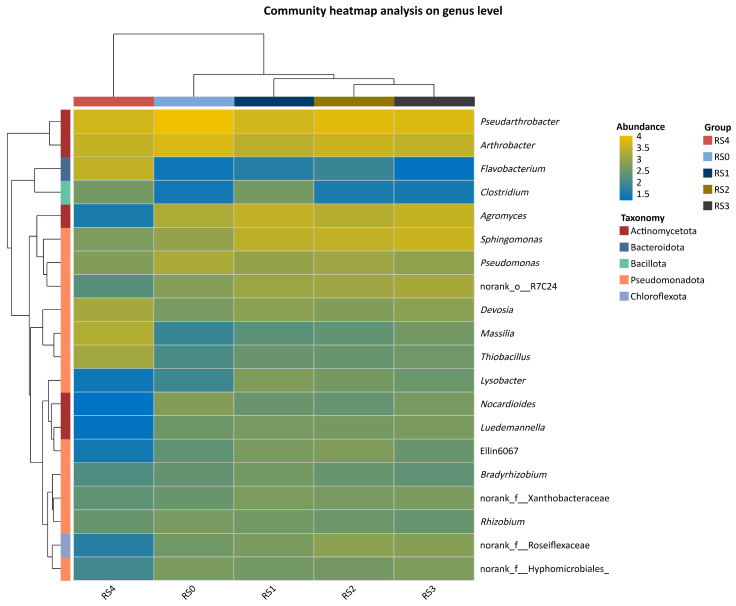
Heatmap and hierarchical clustering of rhizosphere soil bacterial genera across five developmental stages of *GE* (RS0–RS4: initial planting, seedling emergence, bud formation, flowering, and fruiting). Rows represent genera; columns represent stages. Color gradient (yellow, high abundance; blue, low abundance) indicates relative abundance. Hierarchical clustering trees above the heatmap (samples) and to the left (genera) reflect similarity in community composition and abundance correlation among genera, respectively. Top and left color bars denote developmental stage and phylum-level affiliation of each genus, respectively.

Cluster analysis of rhizosphere soil bacterial communities across growth stages revealed significant stage-dependent shifts in species composition. Communities from the initial planting to flowering stages (GS1–GS4) clustered together, with the bud formation (GS3) and flowering (GS4) stages showing the highest similarity. In contrast, the fruiting-stage (GS5) community formed a distinct cluster. Dominant genera such as *Pseudarthrobacter*, *Arthrobacter*, and *Agromyces* remained abundant throughout GS1–GS4, while *Sphingomonas* increased from seedling emergence onward. At the fruiting stage, new dominant genera including *Flavobacterium*, *Massilia*, and *Devosia* emerged. These results indicate that the rhizosphere bacterial community remains relatively stable during early and middle growth stages but undergoes substantial turnover at the fruiting stage (Figure 8).

### 3.4. Persistence and Occurrence–Abundance Patterns of Bacterial Communities

**Figure 9:**
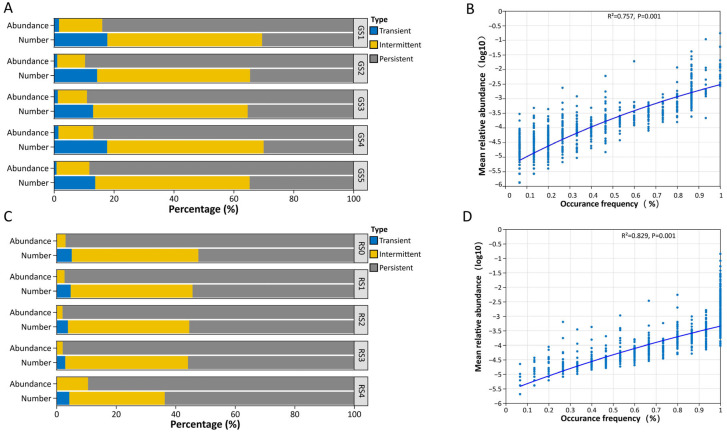
Persistence classification and occurrence–abundance correlation of endophytic and rhizosphere soil bacterial communities across five seed developmental stages (GS1–GS5) of *GE*. (**A**) Stacked bar plot showing the relative proportions (abundance and OTU number) of transient, intermittent, and persistent bacterial taxa in the endophytic community. (**B**) Scatter plot of OTU occurrence frequency versus log_10_-transformed mean relative abundance for endophytic bacteria; the blue line represents linear regression (equation, R^2^, and *p* < 0.001 shown). (**C**) Stacked bar plot as in (**A**) but for the rhizosphere soil community. (**D**) Scatter plot as in (**B**) but for rhizosphere soil bacteria. GS1–GS5: initial planting, seedling emergence, bud formation, flowering, and fruiting stages, respectively. RS0–RS4: rhizosphere soil corresponding to GS1–GS5.

The overall structure of the persistence classification remained stable in different tissues and across stages, with persistent taxa maintaining dominance in abundance throughout seed formation. During the five seed developmental stages of *GE* (GS1–GS5), endophytic bacterial taxa were divided into three persistence types: transient, intermittent and persistent. In terms of relative abundance, persistent taxa dominated the community, contributing more than 80% of the total abundance in different tissues and at each developmental stage. Transient and intermittent taxa contributed only a minor fraction of the total relative abundance (Figure 9A,C and  Figure S11). At the same time, a significant positive correlation was observed between the occurrence frequency and mean relative abundance of bacterial taxa (Figure 9B, R^2^ = 0.780, *p* < 0.001; Figure 9D, R^2^ = 0.829, *p* < 0.001; Figure S12). Taxa with higher occurrence frequency exhibited greater mean relative abundance, which further validated the rationality of the classification into persistent and transient bacterial taxa. These results provide a quantitative basis for identifying the core microbiota and stage-specific taxa of the seed-associated bacterial community.

### 3.5. β-Diversity of Endophytic and Rhizosphere Soil Bacterial Communities

**Figure 10:**
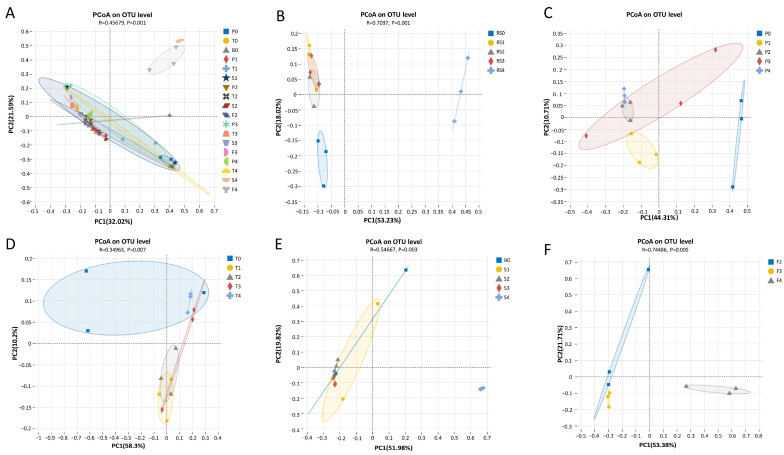
PCoA (principal coordinate analysis) showing endophytic bacterial communities in various tissues and rhizosphere soil bacteria at five developmental stages of *GE* (initial planting, seedling emergence, bud formation, flowering, and fruiting). (**A**) Endophytic bacteria across all stages; (**B**) rhizosphere soil bacteria; (**C**) epidermal endophytic bacteria; (**D**) internal tissue endophytic bacteria; (**E**) stem endophytic bacteria; (**F**) endophytic bacteria in floral stalk, flowers, and seeds. Each point represents an individual sample, with colors/shapes indicating the developmental stage. Proximity between points reflects similarity in community composition. Tissue codes: P0–P4 (epidermis), T0–T4 (internal tissue), B0–S4 (stem), and F2–F4 (floral stalk, flower, seed) corresponding to stages GS1–GS5 as appropriate.

The endophytic bacterial community of *GE* remained relatively stable across most developmental stages and tissues, with significant compositional shifts occurring only at the fruiting stage in specific tissues (seeds, flowers and stems). ANOSIM confirmed high similarity among endophytic communities across stages (R = 0.4568, *p* = 0.001), and PCoA ordination showed substantial overlap among most samples, while stem (S4) and seed (F4) samples at the fruiting stage clustered tightly (Figure 10A). In contrast, rhizosphere soil communities were highly dynamic and stage-sensitive (ANOSIM R = 0.7037, *p* = 0.001). PCoA revealed clear segregation across stages, with the most pronounced divergence between the initial planting (RS0) and fruiting (RS4) stages (Figure 10B).

Tissue-specific analyses further supported these patterns: epidermis communities were similar across emergence to fruiting stages (P1–P4) but diverged at the initial stage (P0; ANOSIM R = 0.5837, *p* = 0.001; Figure 10C); internal tissues showed the highest similarity across all stages, with no significant shifts (ANOSIM R = 0.3496, *p* = 0.007; Figure 10D); stem communities were consistent from initial planting to flowering (B0, S1–S3) but diverged at the fruiting stage (S4; ANOSIM R = 0.5467, *p* = 0.003; Figure 10E); and reproductive tissues exhibited significant differences between fruiting-stage seeds (F4) and flower stalks (F2) or flowers (F3) (ANOSIM R = 0.7449, *p* = 0.005; Figure 10F).

### 3.6. Analysis of Stage-Specific Differentially Enriched Taxa of Bacterial Communities Across Different Developmental Stages

#### 3.6.1. Stage-Specific Differentially Enriched Taxa of Endophytic Bacterial Community

**Figure 11:**
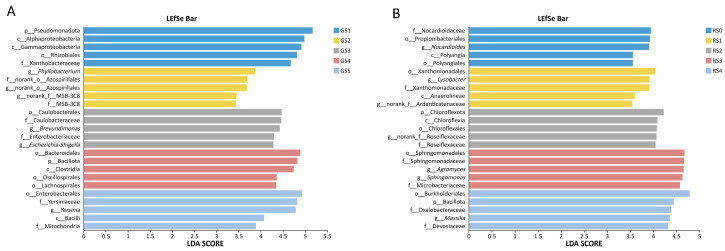
Linear discriminant analysis (LDA) score bar plots from LEfSe showing stage-specific differentially enriched bacterial biomarkers in endophytic and rhizosphere soil communities of *GE* across five seed developmental stages (LDA score threshold ≥ 2.5, *p* < 0.05). Higher LDA scores indicate stronger discriminative power. (**A**) Endophytic community; (**B**) rhizosphere soil community. GS1–GS5: initial planting, seedling emergence, bud formation, flowering, and fruiting stages. RS1–RS4: rhizosphere soil at seedling emergence, bud formation, flowering, and fruiting stages, respectively.

Overall, LEfSe analysis revealed that the seed endophytic bacterial community of *GE* displayed significant stage-specific enrichment patterns across the five developmental stages. Pseudomonadota, particularly Alphaproteobacteria and Rhizobiales, dominated the initial planting stage (GS1) as the core discriminative biomarkers (maximum LDA = 5.17). *Phyllobacterium*-related taxa acted as characteristic biomarkers for the seedling emergence stage (GS2, LDA = 3.43–3.87). In addition, Caulobacterales and Caulobacteraceae were predominant biomarkers at the bud formation stage (GS3, LDA = 4.29–4.47). At the flowering stage (GS4), *Bacteroidetes* and Bacillota became dominant (maximum LDA = 4.9). Finally, Enterobacteriales- and Yersiniaceae-related taxa served as the core biomarkers for the fruiting stage (GS5, LDA = 3.0–4.5) (Figure 11A).

#### 3.6.2. Stage-Specific Differentially Enriched Taxa of Rhizosphere Soil Bacterial Community

Consistent with the seed endophytic community, the rhizosphere soil bacterial community of *GE* exhibited significant stage specificity across the five growth phases. The initial planting stage (RS0) was enriched with Nocardioidaceae and *Nocardioides*-related taxa (maximum LDA = 3.94). The seedling emergence stage (RS1) harbored abundant Xanthomonadaceae- and *Lysobacter*-related taxa (LDA = 3.54–4.04). At the bud formation stage (RS2), *Chloroflexi* and affiliated lineages (Chloroflexota, Chloroflexales, and Roseiflexaceae) showed absolute dominance (LDA = 4.05–4.22). During the flowering stage (RS3), Sphingomonadales, Sphingomonadaceae, and *Sphingomonas* were significantly enriched (maximum LDA = 4.68). Finally, at the fruiting stage (RS4), Burkholderiales and Bacillota became dominant (LDA = 4.31–4.79) (Figure 11B).

#### 3.6.3. Stage-Specific Differentially Enriched Bacterial Taxa Across Different Tissue Compartments

A total of 128 differentially enriched bacterial taxa were identified across all tissue compartments, each exhibiting significant discriminative power for distinguishing tissue- and stage-specific community characteristics (Figures S13 and S14). Specifically, at the initial planting stage, the epidermis (P0) was dominated by Gammaproteobacteria, particularly Pseudomonadales and *Pseudomonas* (maximum LDA = 5.06). In stem tissues covering successive developmental stages (S1–S4), Elsterales and Enterobacterales served as key differential biomarkers (maximum LDA = 5.42). At the seedling emergence stage, the seed internal tissue (T1) was enriched in Hyphomonadaceae, *Nakamurella*, and Cellvibrionales (maximum LDA = 3.31). Finally, in reproductive tissues (floral stalks and flowers, F2–F3), Actinomycetota, Butyricicoccaceae and Mitochondria served as core discriminative biomarkers (maximum LDA = 4.52) (Figure S14).

### 3.7. Functional Prediction of Bacterial Communities

**Figure 12:**
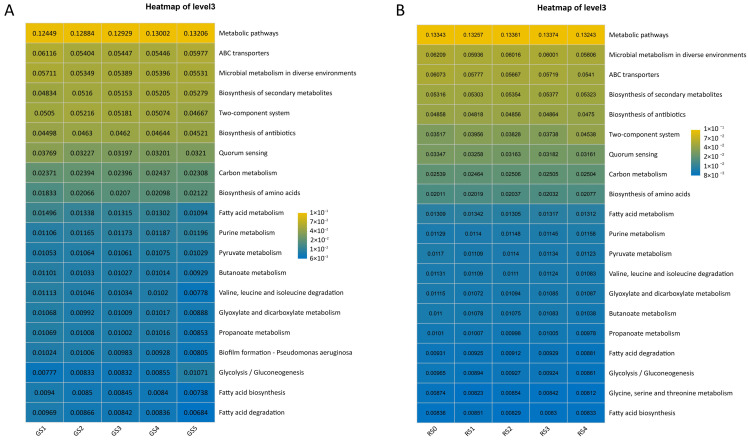
Heatmap of predicted KEGG level 3 metabolic pathways (Tax4Fun) for endophytic (**A**) and rhizosphere soil (**B**) bacterial communities across five seed developmental stages of *GE* (GS1–GS5: initial planting to fruiting; RS0–RS4: corresponding rhizosphere soils). Color gradient (yellow, high abundance; blue, low abundance) indicates relative abundance of the top 20 pathways.

Both endophytic and rhizosphere bacterial communities displayed stable core functional profiles during seed formation, with minor stage-specific shifts in secondary metabolic and signaling pathways reflecting host–microbe interactions. In endophytic communities, core pathways (e.g., metabolic pathways, ABC transporters, secondary metabolite biosynthesis, and two-component system) were consistently abundant across all stages. Stage-specific patterns included the enrichment of ABC transporters at the initial planting stage (GS1), a progressive increase in carbon metabolism and amino acid biosynthesis from GS1 to flowering (GS4), and significant enrichment of glycolysis or gluconeogenesis at the flowering and fruiting (GS5) stages (Figure 12A).

Rhizosphere communities shared similar core pathways but exhibited more pronounced stage specificity, consistent with their higher structural sensitivity. Basal metabolism and environmental adaptation pathways were enriched at the initial planting stage (RS0), whereas glycolysis or gluconeogenesis and fatty acid metabolism pathways were significantly enriched at late stages (RS3–RS4) (Figure 12B).

## 4. Discussion

*Gastrodia elata* Blume (*GE*) is a high-value traditional Chinese medicinal herb that supports the economic development of major producing regions in China, including Yunnan, Guizhou, and Hubei Provinces [17,41,42,43]. Investigating the endophytic and rhizosphere bacterial communities of *GE* is critical for optimizing its cultivation, improving yield and quality, and promoting sustainable production practices. While recent advances in microbiome technology have expanded research on *GE*-associated microbial communities, existing studies have predominantly focused on tuber endophytic fungi or rhizosphere microorganisms during the vegetative growth stage [11,14,15,44]. A critical research gap remains regarding the spatiotemporal dynamics of endophytic and rhizosphere bacterial communities throughout the sexual reproductive phase of *GE*, particularly during seed formation and maturation. High-throughput 16S rRNA gene sequencing (HTS) has emerged as a powerful tool for profiling endophytic bacterial communities, enabling unprecedented insights into their diversity and potential functions [45]. Accordingly, this study systematically analyzed the composition, diversity, and spatiotemporal succession patterns of endophytic and rhizosphere soil bacterial communities across five critical stages of *GE* seed formation (initial planting, seedling emergence, bud formation, flowering, and fruiting) using HTS technology. The findings presented here fill the research gap in bacterial community dynamics during the seed formation stages of *GE* and provide novel insights into the mechanisms of plant–microbe interactions in this mycoheterotrophic orchid.

Orchidaceae plants typically harbor diverse endophytic microbial communities [46,47], with endophytic fungi having well-established roles in seed germination and nutrient provision, while the functional exploration of endophytic bacteria remains in its early stages [5,46,48]. The dominant endophytic bacterial phyla identified in most orchid species include Pseudomonadota, Actinobacteria and Bacteroidota, which aligns with the predominant phyla observed in the endophytic bacterial communities of *GE* in this study, and also agrees with previous findings on *Platanthera praeclara* [49], *Dendrobium* spp. [50] and *Neottia ovata* [3]. However, significant variations exist among orchid species in terms of dominant genera and community dynamics: *Dendrobium* spp. commonly harbor high abundances of *Pseudomonas*, *Burkholderia*, and *Bacillus*, while *Neottia ovata* is enriched in *Pseudoxanthomonas*, *Rhizobium*, and *Mitsuaria*.

In this study, we observed a high relative abundance of *Bacteroides* in the aerial tissues of *GE* at the flowering stage. It remains atypical for above-ground plant compartments, as *Bacteroides* are predominantly recognized as anaerobic gut- or soil-associated bacteria. To critically address concerns regarding potential environmental contamination or primer bias, we performed systematic technical validations. All tissue samples were handled under strict aseptic conditions with a verified surface sterilization protocol. No signals of *Bacteroides* were found in no-template PCR controls, environmental blanks and surface rinse controls, which effectively excluded exogenous contamination. Furthermore, the universal 16S rRNA primers employed have been widely validated for plant endophytic microbiome research [51,52], and our sequencing data underwent rigorous quality filtering and multi-database taxonomic annotation, effectively eliminating primer bias as a confounding factor. From a biological perspective, this enrichment may be attributed to the robust carbohydrate-degrading capacity of *Bacteroides*, which could support the energy-intensive reproductive development of this mycoheterotrophic *GE*.

It should be noted that this result diverges somewhat from the research conducted by Zheng et al. on endophytic bacteria in *GE* tubers, with distinct Pseudomonadota and Acidobacteriota as the dominant phyla and with community structures varying across production areas [14]. This difference may be attributed to the stage and tissue specificity of *GE* endophytic bacterial communities: previous studies focused on under-ground tubers during the vegetative growth stage, whereas our work focused on above-ground tissues during sexual development stages.

Furthermore, endophytic bacteria in *GE* exhibited distinct stage-specific succession. The dominant genera shifted from *Pseudomonas* and *Brevundimonas* to *Bacteroides* and *Escherichia-Shigella*, with the abundance of *Bacteroides* slightly decreasing at the fruiting stage. This dynamic shift may be closely linked to the physiological state and metabolic demands of *GE* across different developmental stages. For example, *Pseudomonas* species are commonly found in the endosphere of plants and may contribute to growth promotion and disease resistance during the early vegetative growth stage [53,54], while the enrichment of *Bacteroides* during the reproductive stage may be associated with the changing nutritional requirements of developing seeds.

Rhizosphere soil bacterial communities are influenced by a range of abiotic factors, including soil type and climate, and are also closely associated with the developmental stage of the host plant, as root exudation and plant physiological status change dynamically throughout growth [55,56,57]. In this study, the dominant phyla of rhizosphere soil bacteria were Pseudomonadota and Actinomycetota, with *Pseudarthrobacter* and *Arthrobacter* consistently present across all stages of *GE* seed formation. These two genera are widely distributed in soil environments and are known for their capacity to decompose organic matter and mediate nutrient cycling [58], which may play a potential role in maintaining rhizosphere microecological balance during *GE* seed formation.

Khanh et al. previously investigated rhizosphere soil bacteria during the vegetative growth stage of *GE*, reporting Pseudomonadota, Acidobacteriota, and *Bacteroidetes* as the dominant phyla and observing a gradual decrease in the number of rhizosphere bacterial genera as *GE* grew, with some core strains remaining stable throughout the growth stages [15]. In contrast, during seed development, the rhizosphere soil of *GE* was predominated by Pseudomonadota and Actinomycetota. Bacterial genera increased obviously, core microbial groups remained stable, and novel dominant taxa appeared at the seed maturation stage. These genera often participate in organic matter degradation and nutrient cycling [59,60,61], a process that may be linked to changes in the composition of root exudates during *GE* fruit ripening. These findings are partially aligned with the observations made by Khanh et al., particularly regarding the stability of certain core bacterial genera throughout growth, but explicitly highlight the distinct differences in rhizosphere bacterial community dynamics between the vegetative and reproductive growth stages of *GE*.

Endophytic bacterial communities in different tissues of *GE* showed obvious spatiotemporal differentiation. We observed that during the initial planting stage, significant differences in bacterial composition existed among various tissues; however, from the seedling emergence stage to the flowering stage, the community structures across different tissues became increasingly similar. This convergence may be attributed to the coordinated metabolic processes of *GE* during the early seed formation stages, which may create a more consistent internal microenvironment across tissues, thereby selecting for similar endophytic bacterial communities. By the fruiting stage, seeds and stems formed independent, distinct clusters, indicating that these tissues developed unique microenvironments to meet their specific physiological needs during seed maturation. These results indicate that during reproductive growth, *GE* can selectively enrich specific bacteria via internal metabolism and signal regulation to promote seed development and maturation. This discovery is consistent with studies on other Orchidaceae plants such as *Dendrobium* spp. [8] and *Vanilla planifolia* [9], which also reported significant differences in endophytic bacterial communities among different tissue parts, a phenomenon that may be related to the accumulation of secondary metabolites and the specific physiological functions of each tissue.

Analysis of α- and β-diversity revealed that the endophytic bacterial community of *GE* exhibited peak diversity during the flowering stage and maintained consistent community stability across most developmental stages. This finding contrasts with conclusions drawn in some studies on Orchidaceae plants, where bacterial communities were observed to undergo significant reorganization throughout growth stages [2,62]. For instance, endophytic bacterial communities of autotrophic and partially heterotrophic Orchidaceae undergo obvious structural rearrangement during the transition from vegetative to reproductive growth [3,63]. The relative stability of the endophytic bacterial community in *GE* during seed formation may be related to its unique obligately heterotrophic lifestyle: since *GE* relies entirely on symbiotic fungi for carbon and nutrient supply, the endophytic bacterial community may not need to undergo significant reorganization to adapt to changes in host nutrient sources. During the fruiting stage, *GE* may maintain a relatively stable core endophytic bacterial community, which may contribute continuously to the host’s physiological balance, such as maintaining cell homeostasis and promoting nutrient transport for seed development.

In contrast, the α-diversity of rhizosphere soil bacteria peaked during the bud formation stage, which may be due to the increased release of root exudates during bud development, providing more carbon sources and nutrients for rhizosphere bacteria and thereby promoting the growth and reproduction of diverse bacterial groups. β-Diversity analysis revealed that the rhizosphere soil community composition changed more markedly with developmental stages, suggesting that the rhizosphere soil bacterial community was particularly responsive to environmental fluctuations and plant growth phases. This finding aligns with the conclusions of Chaparro et al. and Chen et al. regarding the “recombination of the rhizosphere microbiome with plant growth stages” [64,65], further highlighting the dynamic regulatory role of the rhizosphere microecosystem throughout plant development. The dynamic changes in rhizosphere bacterial communities during *GE* seed formation may represent an adaptive strategy of the plant to optimize nutrient acquisition and environmental adaptation during the critical reproductive stage.

Several limitations of this study need to be acknowledged. First, the functions of core dominant bacteria were only predicted via taxonomic annotation and bioinformatics analysis, without validation by in vitro and in vivo functional experiments. Second, this study was conducted in an indoor artificial cultivation environment, which differs from the natural field environment in terms of soil conditions, climate, and biotic interactions; these differences may have confounding effects on the composition of bacterial communities, and the ecological relevance of the results to natural field-grown *GE* requires further validation. Third, key differentially enriched bacterial strains were not isolated and cultured; hence, their specific functions in seed formation of *GE* still remain unclarified.

In summary, this study systematically analyzed the dynamic changes of endophytic and rhizosphere soil bacterial communities during the seed formation stage of *GE*, revealing the stage-specific successional dynamics and tissue-specific characteristics of bacterial communities. The findings indicate that the endophytic and rhizosphere soil bacterial communities of *GE* are closely associated with its reproductive growth and seed development and may play potential roles in nutrient cycling, physiological regulation, and seed maturation. Future research should focus on isolating and identifying key functional bacterial strains, verifying their roles in *GE* seed formation through pot experiments or in vitro culture, and exploring the molecular mechanisms of plant–microbe interactions. This will provide a more solid theoretical basis for the optimization of *GE* cultivation techniques, for the improvement of seed quality, and fostering sustainable growth in the *GE* industry.

## 5. Conclusions

This study systematically characterized the spatiotemporal dynamics of endophytic and rhizosphere soil bacterial communities across five seed formation stages of *Gastrodia elata* Blume (*GE*) using high-throughput 16S rRNA sequencing. The core finding is that both bacterial compartments exhibit significant stage- and tissue-specific successional dynamics while maintaining a stable core microbiome linked to host reproductive development. Endophytic bacterial communities were relatively stable across most developmental stages and tissues, and obvious compositional variations were only detected in seeds and stems at the fruiting stage. Diversity and richness peaked at flowering stage, and *Bacteroides* served as the core persistent genus. In contrast, rhizosphere communities were far more dynamic and stage-sensitive, with substantial turnover at the fruiting stage, and *Pseudarthrobacter* was identified as the core persistent genus. This work fills a critical gap by being the first to systematically resolve the spatiotemporal successional dynamics of both endophytic and rhizosphere bacterial communities throughout the entire seed formation cycle of a mycoheterotrophic orchid, expanding the theoretical framework of plant–microbe interactions during reproductive development.

## Figures and Tables

**Table 1:** Indices of diversity and richness of bacterial communities at distinct stages.

Index		Initial Planting (GS1)	Seeding Emergence (GS2)	Bud Formation (GS3)	Flowering (GS4)	Fruiting (GS5)
Shannon	EB	3.97 ± 0.98 bc	4.77 ± 0.67 ab	4.85 ± 0.61 ab	5.14 ± 0.28 a	3.48 ± 2.12 c
	RB	5.22 ± 0.35 a	5.22 ± 0.36 a	5.40 ± 0.02 a	5.37 ± 0.12 a	5.23 ± 0.18 a
Simpson	EB	0.069 ± 0.042 b	0.033 ± 0.038 b	0.031 ± 0.033 b	0.019 ± 0.005 b	0.249 ± 0.284 a
	RB	0.032 ± 0.016 a	0.032 ± 0.017 a	0.019 ± 0.002 ab	0.019 ± 0.003 ab	0.020 ± 0.004 ab
Ace	EB	697.42 ± 402.58 a	958.2 ± 559.08 a	829.18 ± 432.23 a	1113.83 ± 484.59 a	841.73 ± 276.17 a
	RB	3462.26 ± 325.52 a	3462.26 ± 325.53 a	3502.95 ± 89.25 a	3174.73 ± 218.03 a	2539.04 ± 205.51 b
Chao	EB	674.94 ± 427.71 a	861.11 ± 486.99 a	780.99 ± 329.87 a	1105.84 ± 446.54 a	835.20 ± 314.78 a
	RB	3036.91 ± 166.14 a	3036.91 ± 166.15 a	3218.52 ± 78.16 a	2945.33 ± 153.08 a	2363.79 ± 173.37 b
Coverage	EB	0.9965 ± 0.00220 a	0.9954 ± 0.00393 a	0.9969 ± 0.00259 a	0.9948 ± 0.00469 a	0.9958 ± 0.00097 a
	RB	0.9733 ± 0.00141 b	0.9733 ± 0.00142 b	0.9709 ± 0.00105 b	0.9740 ± 0.00169 b	0.9799 ± 0.00170 a

Note: In the same row, data marked with different lowercase letters indicate significant differences across stages (*p* < 0.05). Here, EB denotes endophytic bacteria and RB denotes rhizosphere soil bacteria.

## Data Availability

Data are available from the authors upon request.

## Supplementary Materials

The following supporting information can be downloaded at https://www.mdpi.com/article/10.3390/biology15110829/s1, Figure S1: Rarefaction curves of OTU of endophytic bacteria in tissues and rhizosphere soil bacteria of *GE* at different developmental stages; Figure S2: Phylum-level taxonomic composition of endophytic bacterial communities across five seed developmental stages (GS1–GS5) of *GE*; Figure S3: Community analysis pie plot of endophytic bacteria in different tissues at different stages of *GE* at phylum level; Figure S4: Community analysis pie plot of endophytic bacteria from *GE* at different developmental stages at genus level; Figure S5: Stacked bar plot of endophytic bacterial relative abundance at the phylum level in different tissues across five seed developmental stages (GS1–GS5) of *GE*; Figure S6: Community analysis pie plot of endophytic bacteria in different tissues at different stages of *GE* at phylum level; Figure S7: Community analysis pie plot of endophytic bacteria at the genus level in different tissues at different stages of *GE*; Figure S8: Phylum-level taxonomic composition of rhizosphere soil bacterial communities across five seed developmental stages of *GE*; Figure S9: Community analysis pie plot of rhizospheric soil bacteria from *GE* at different developmental stages at phylum level; Figure S10: Community analysis pie plot of rhizospheric soil bacteria from *GE* at different developmental stages at genus level; Figure S11: Temporal dynamic distribution patterns of bacterial communities across different tissue compartments and developmental stages of *GE* during seed formation; Figure S12: Occupancy–Abundance Distribution analysis of bacterial communities across different tissue compartments and developmental stages of *GE* during seed formation; Figure S13: Linear discriminant analysis (LDA) score bar plot from LEfSe analysis, showing the discriminative power of differentially enriched bacterial biomarkers across different tissues and seed developmental stages of *GE*; Figure S14: Linear discriminant analysis (LDA) score bar plot from LEfSe analysis. Table S1: Sample grouping and coding scheme for *GE* across five seed formation developmental stages; Table S2: OTUs of endophytic bacteria and rhizosphere soil bacteria from *GE* at different developmental stages; Table S3: OTUs of endophytic bacteria from *GE* at different developmental stages; Table S4: OTUs of rhizosphere soil bacteria from *GE* at different developmental stages; Table S5: Diversity and richness indices of endogenous bacterial communities in the tissues of *GE* at different developmental stages; Table S6: Diversity and richness indices of endophytic bacterial communities in the epidermis at different developmental stages of *GE*; Table S7: Diversity and richness indices of endophytic bacterial communities in the tissues of *GE* at different developmental stages; Table S8: Diversity and richness indices of endophytic bacterial communities in the stems of *GE* at different developmental stages; Table S9: Diversity and richness indices of endophytic bacterial communities in the floral stalks, flowers and seeds of *GE*.
