# Supplementary material for: Community Succession and Diversity Variation of Endophytic and Rhizosphere Soil Bacteria Across Gastrodia elata Seed Formation Stages
Source: Biology (Basel). 2026 May 25;15(11):829. doi: 10.3390/biology15110829 (PMC13255848; doi:10.3390/biology15110829)
Supplement: Supplementary file 1 [file biology-15-00829-s001.zip › Figure S1 Rarefaction curves of OTU of endophytic bacteria in tissues and rhizosphere soil bacteria of G. elata at different developmental stages.pdf]

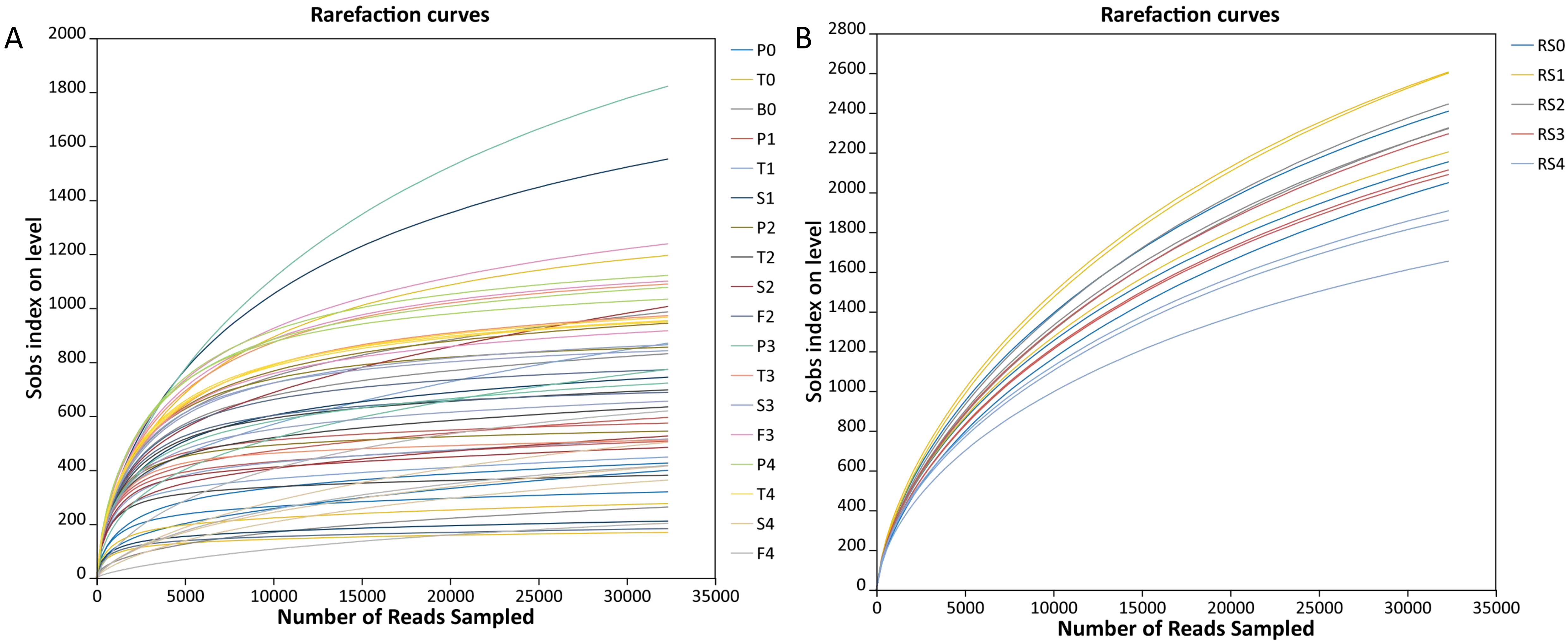

Figure S1. Rarefaction curves of OTU of endophytic bacteria in tissues and rhizosphere soil bacteria of *G. elata* at different developmental stages. (A) Rarefaction curves for the endophytic bacterial community across different tissue compartments. (B) Rarefaction curves for the rhizosphere soil bacterial community across five seed developmental stages. The x-axis represents the number of randomly sampled sequencing reads, and the y-axis represents the Sobs index, which reflects the observed number of bacterial operational taxonomic units (OTUs) in the sample. P0-P4 represent the epidermis, T0-T4 represent the internal tissue and S0-S4 represent the stem tissue. The tissue codes listed above correspond to five seed developmental stages: the initial planting (GS1), seeding emergence (GS2), bud formation (GS3), flowering (GS4), and fruiting (GS5) stages, respectively. F2-F4 represent the floral bud stalk, flower and seed tissue; RS0: The rhizosphere soil of initial planting, RS1: The rhizosphere soil of seeding emergence, RS2: The rhizosphere soil of bud formation, RS3: The rhizosphere soil of flowering, RS4: The rhizosphere soil of fruiting.
