# Supplementary material for: Community Succession and Diversity Variation of Endophytic and Rhizosphere Soil Bacteria Across Gastrodia elata Seed Formation Stages
Source: Biology (Basel). 2026 May 25;15(11):829. doi: 10.3390/biology15110829 (PMC13255848; doi:10.3390/biology15110829)
Supplement: Supplementary file 1 [file biology-15-00829-s001.zip › Figure S11. Temporal dynamic distribution patterns of bacterial communities across different tissue compartments and developmental stages of GE during seed formation.pdf]

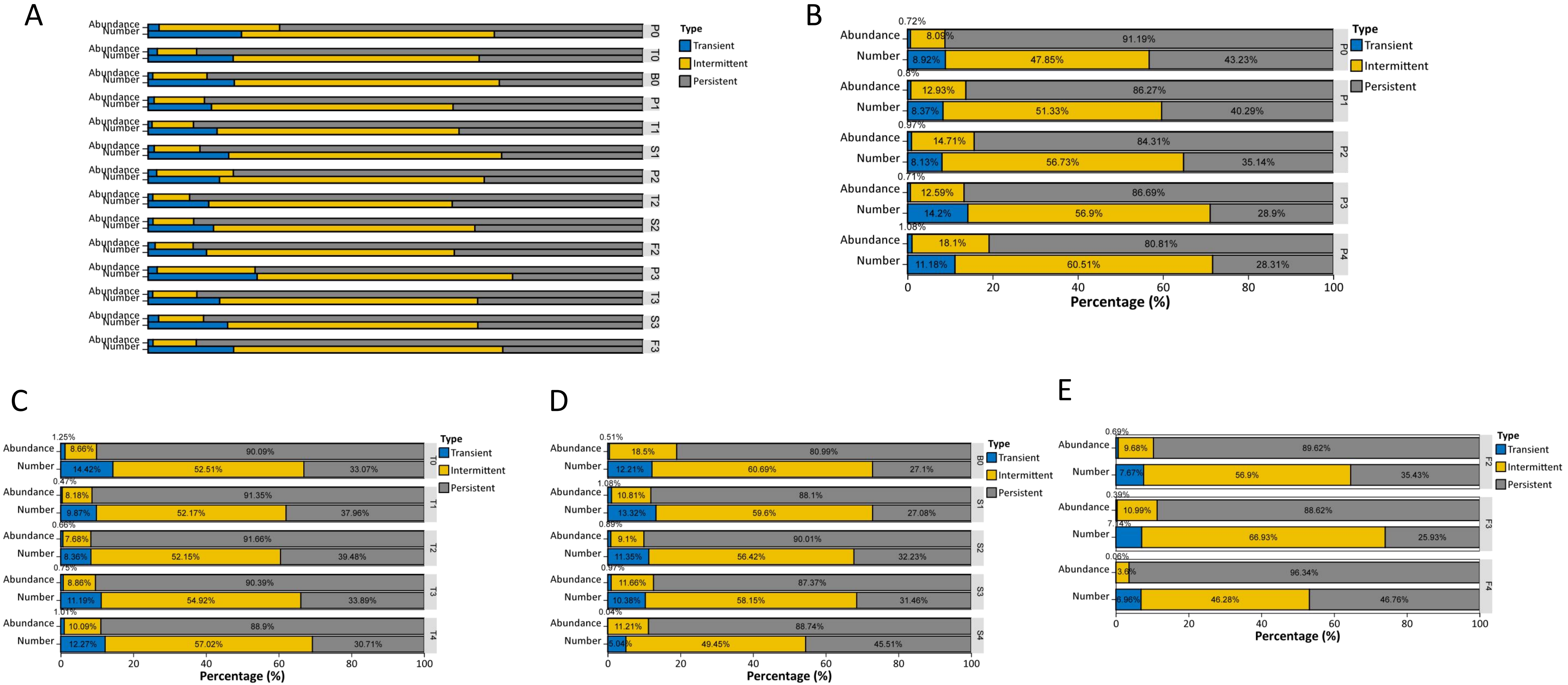

Figure S11. Temporal dynamic distribution patterns of bacterial communities across different tissue compartments and developmental stages of *GE* during seed formation. (A) Overview of the temporal dynamic distribution of bacterial communities across all tissue types and developmental stages. (B) Temporal dynamic distribution of bacterial communities in epidermis at five seed developmental stages. (C) Temporal dynamic distribution of bacterial communities in internal tissue across the five seed developmental stages. (D) Temporal dynamic distribution of bacterial communities in stem tissue at the five seed developmental stages stages. (E) Temporal dynamic distribution of bacterial communities in reproductive tissue. P0-P4 represent the epidermis, T0-T4 represent the internal tissue and S0-S4 represent the stem tissue. The tissue codes listed above correspond to five seed developmental stages: the initial planting (GS1), seeding emergence (GS2), bud formation (GS3), flowering (GS4), and fruiting (GS5) stages, respectively. F2-F4 represent the floral bud stalk, flower and seed tissue.
