# Supplementary material for: Community Succession and Diversity Variation of Endophytic and Rhizosphere Soil Bacteria Across Gastrodia elata Seed Formation Stages
Source: Biology (Basel). 2026 May 25;15(11):829. doi: 10.3390/biology15110829 (PMC13255848; doi:10.3390/biology15110829)
Supplement: Supplementary file 1 [file biology-15-00829-s001.zip › Figure S12. Occupancy-Abundance Distribution analysis of bacterial communities across different tissue compartments and developmental stages of GE during seed formation.pdf]

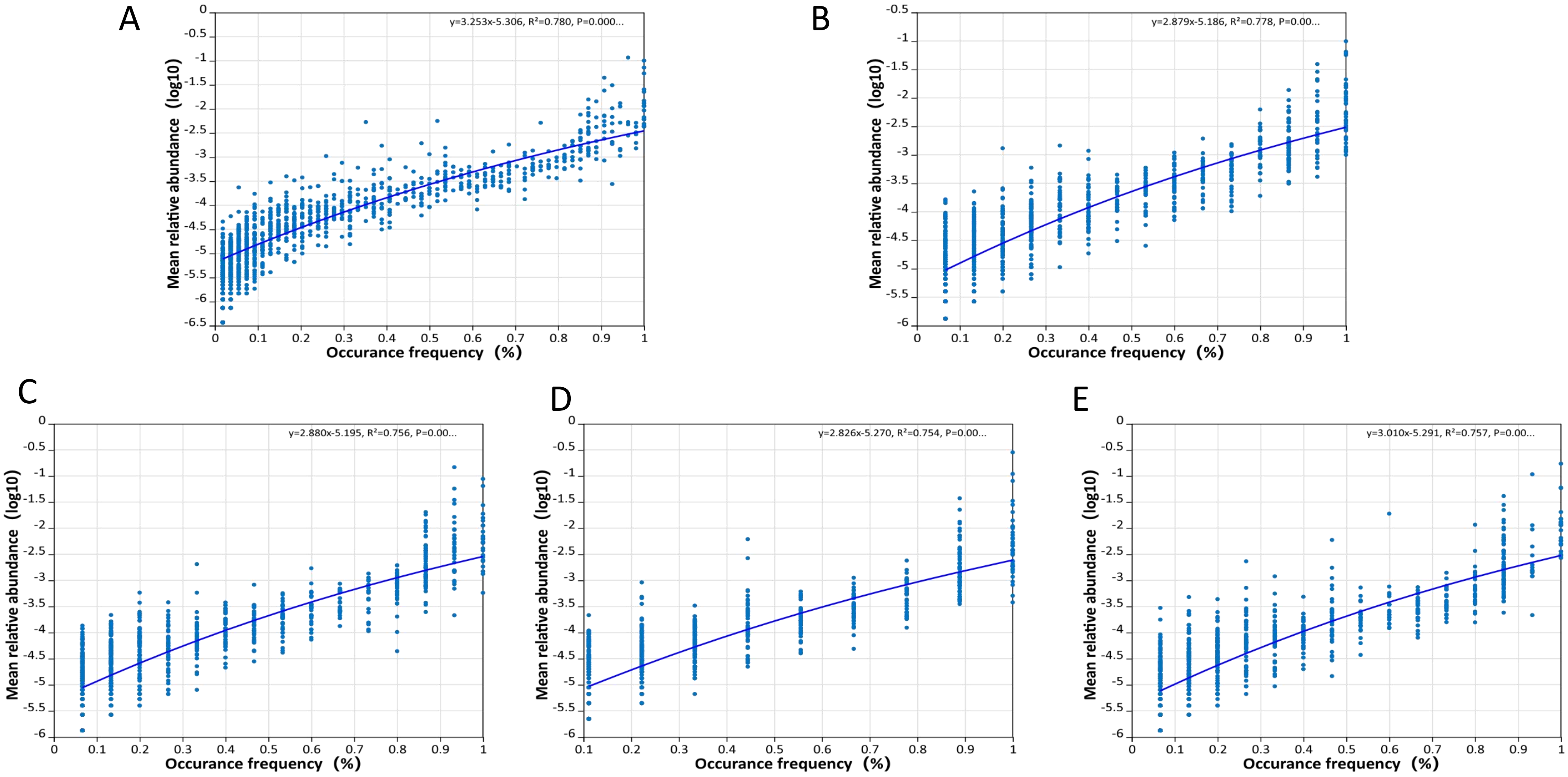


**Figure S12.** Occupancy-Abundance Distribution analysis of bacterial communities across different tissue compartments and developmental stages of *GE* during seed formation. (A) Occupancy-Abundance Distribution of bacterial communities across all tissue types and developmental stages of *G. elata*. (B) Occupancy-Abundance Distribution of bacterial communities in in epidermis at five seed developmental stages. (C) Occupancy-Abundance Distribution of bacterial communities in internal tissue across the five seed developmental stages. (D) Occupancy-Abundance Distribution of bacterial communities in stem tissue at the five seed developmental stages stages. (E) Occupancy-Abundance Distribution of bacterial communities in reproductive tissue.
