# Supplementary material for: Community Succession and Diversity Variation of Endophytic and Rhizosphere Soil Bacteria Across Gastrodia elata Seed Formation Stages
Source: Biology (Basel). 2026 May 25;15(11):829. doi: 10.3390/biology15110829 (PMC13255848; doi:10.3390/biology15110829)
Supplement: Supplementary file 1 [file biology-15-00829-s001.zip › Figure S13. Linear discriminant analysis (LDA) score bar plot from LEfSe analysis, showing the discriminative powe.pdf]

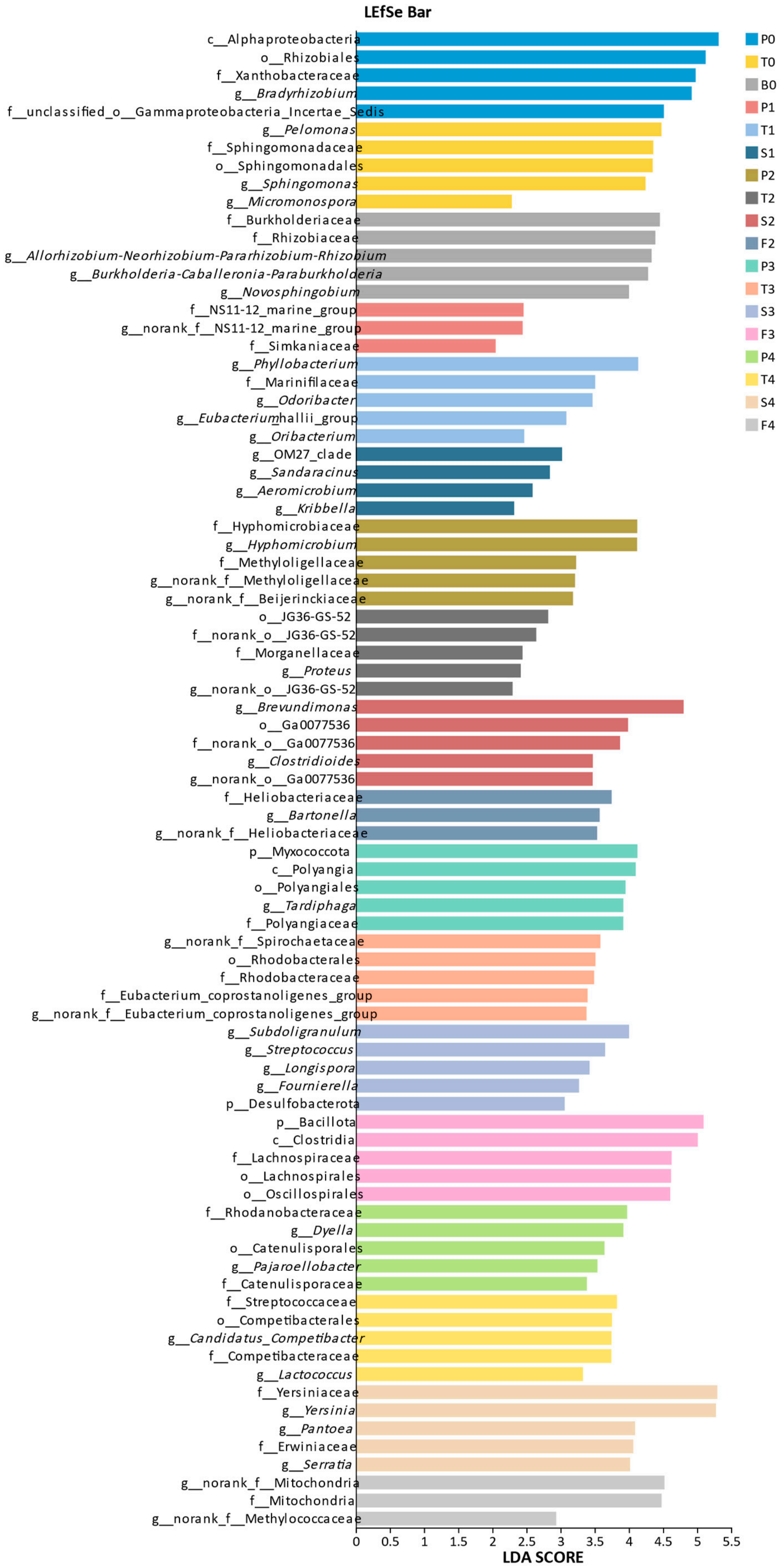
LEfSe Bar
c__Alphaproteobacteria
o__Rhizobiales
f__Xanthobacteraceae
g__Bradyrhizobium
f__unclassified_o__Gammaproteobacteria_Incertae_Sedis
g__Pelomonas
f__Sphingomonadaceae
o__Sphingomonadales
g__Sphingomonas
g__Micromonospora
f__Burkholderiaceae
f__Rhizobiaceae
g__Allorhizobium-Neorhizobium-Pararhizobium-Rhizobium
g__Burkholderia-Caballeronia-Paraburkholderia
g__Novosphingobium
f__NS11-12_marine_group
g__norank_f__NS11-12_marine_group
f__Simkaniaceae
g__Phyllobacterium
f__Marinifilaceae
g__Odoribacter
g__Eubacterium_hallii_group
g__Oribacterium
g__OM27_clade
g__Sandaracinus
g__Aeromicrobium
g__Kribbella
f__Hyphomicrobiaceae
g__Hyphomicrobium
f__Methyloligellaceae
g__norank_f__Methyloligellaceae
g__norank_f__Beijerinckiaceae
o__JG36-GS-52
f__norank_o__JG36-GS-52
f__Morganellaceae
g__Proteus
g__norank_o__JG36-GS-52
g__Brevundimonas
o__Ga0077536
f__norank_o__Ga0077536
g__Clostridioides
g__norank_o__Ga0077536
f__Heliobacteriaceae
g__Bartonella
g__norank_f__Heliobacteriaceae
p__Myxococcota
c__Polyangia
o__Polyangiales
g__Tardiphaga
f__Polyangiaceae
g__norank_f__Spirochaetaceae
o__Rhodobacterales
f__Rhodobacteraceae
f__Eubacterium_coprostanoligenes_group
g__norank_f__Eubacterium_coprostanoligenes_group
g__Subdoligranulum
g__Streptococcus
g__Longispora
g__Fournierella
p__Desulfobacterota
p__Bacillota
c__Clostridia
f__Lachnospiraceae
o__Lachnospirales
o__Oscillospirales
f__Rhodanobacteraceae
g__Dyella
o__Catenulisporales
g__Pajaroellobacter
f__Catenulisporaceae
f__Streptococcaceae
o__Competibacterales
g__Candidatus_Competibacter
f__Competibacteraceae
g__Lactococcus
f__Yersiniaceae
g__Yersinia
g__Pantoea
f__Erwiniaceae
g__Serratia
g__norank_f__Mitochondria
f__Mitochondria
g__norank_f__Methylococcaceae
0
0.5
1
1.5
2
2.5
3
3.5
4
4.5
5
5.5
LDA SCORE
P0
T0
B0
P1
T1
S1
P2
T2
S2
F2
P3
T3
S3
F3
P4
T4
S4
F4

Figure S13. Linear discriminant analysis (LDA) score bar plot from LEfSe analysis, showing the discriminative power of differentially enriched bacterial biomarkers across different tissues and seed developmental stages of *GE* (LDA score threshold ⩾2.5, $P<0.05$). Higher LDA scores indicate stronger discriminative power. Tissue codes: P0–P4 (epidermis), T0–T4 (internal tissue), S0–S4 (stem), and F2–F4 (floral bud stalk, flower, seed), corresponding to stages GS1–GS5 as appropriate.
