# Supplementary material for: Community Succession and Diversity Variation of Endophytic and Rhizosphere Soil Bacteria Across Gastrodia elata Seed Formation Stages
Source: Biology (Basel). 2026 May 25;15(11):829. doi: 10.3390/biology15110829 (PMC13255848; doi:10.3390/biology15110829)
Supplement: Supplementary file 1 [file biology-15-00829-s001.zip › Figure S14. Linear discriminant analysis (LDA) score bar plot from LEfSe analysis.pdf]

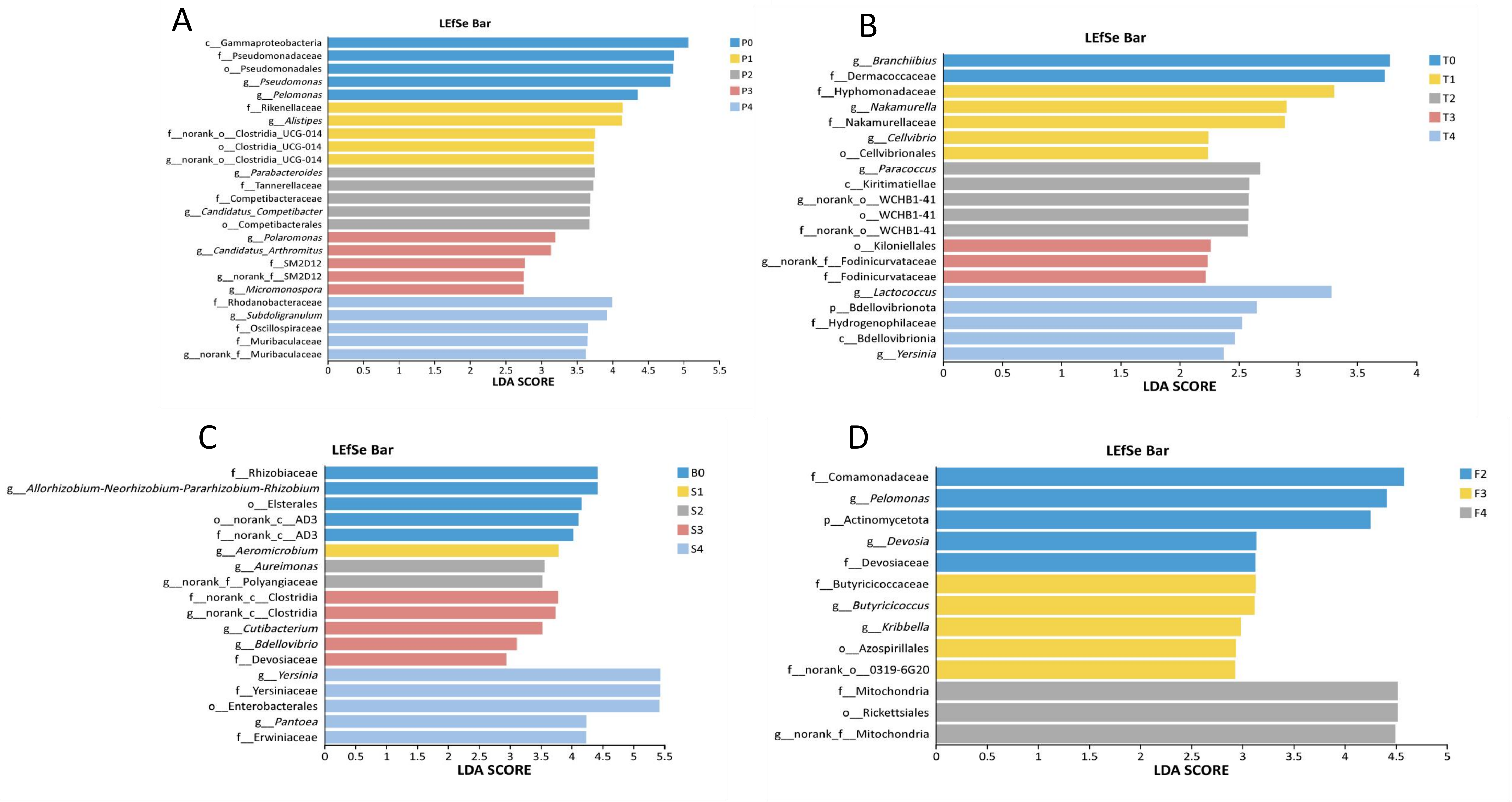

Figure S14. Linear discriminant analysis (LDA) score bar plot from LEfSe analysis, showing the discriminative power of differentially enriched bacterial biomarkers across different tissue compartments and developmental stages of GE seeds. Tissue codes P0–P4, T0–T4, and S0–S4 correspond to the five developmental stages (initial planting to fruiting). Codes F2–F4 correspond to bud formation, flowering, and fruiting stages, respectively.
