# Supplementary material for: Community Succession and Diversity Variation of Endophytic and Rhizosphere Soil Bacteria Across Gastrodia elata Seed Formation Stages
Source: Biology (Basel). 2026 May 25;15(11):829. doi: 10.3390/biology15110829 (PMC13255848; doi:10.3390/biology15110829)
Supplement: Supplementary file 1 [file biology-15-00829-s001.zip › Figure S2.Phyum‐level taxonomic composition of endophytic bacterial communities across five seed developmenta lstages (GS1–GS5) of GE.pdf]

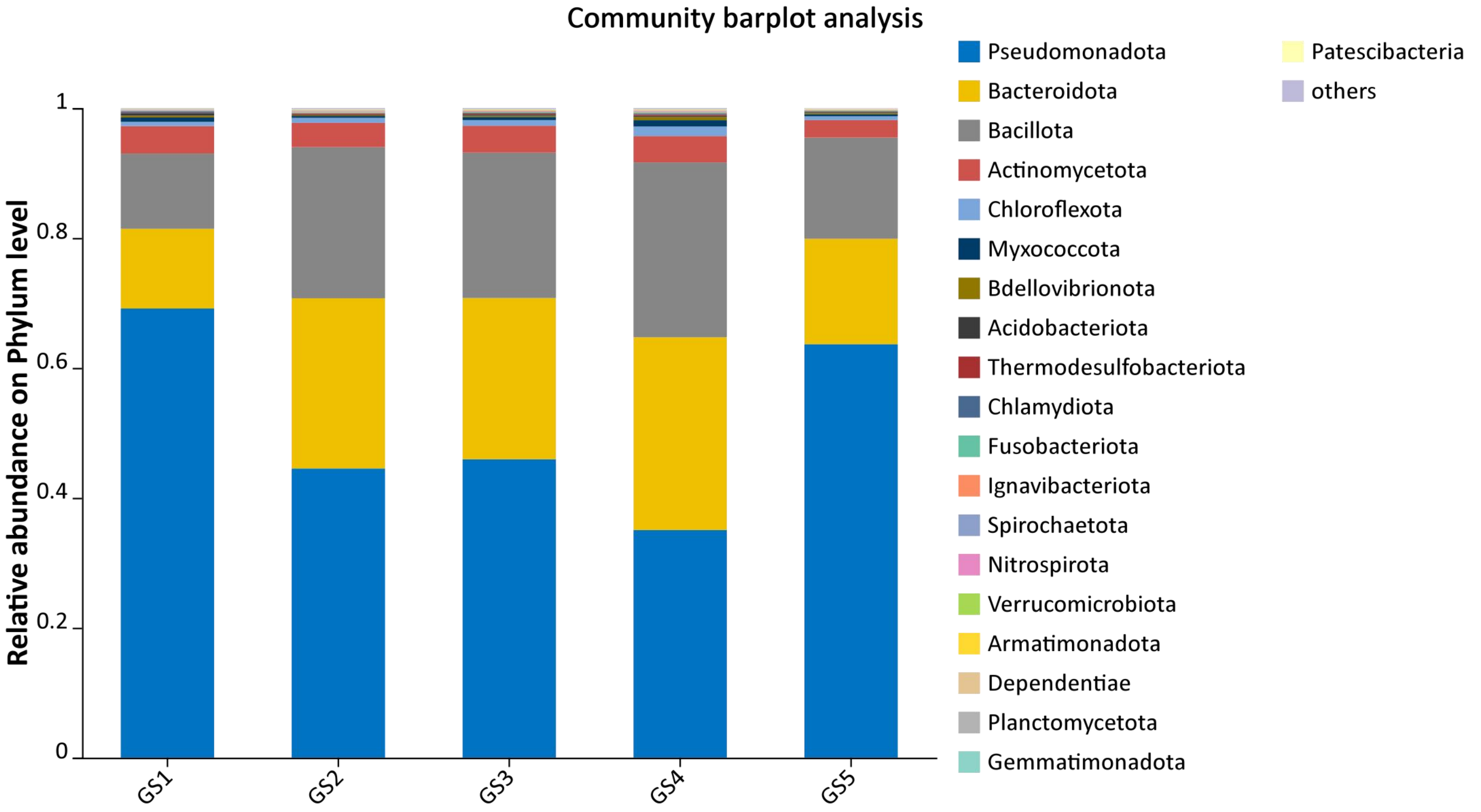

Figure S2. Phyum-level taxonomic composition of endophytic bacterial communities across five seed developmental stage s(GS1–GS5) of *GE*. GS1: initialplanting; GS2: seedling emergence; GS3: bud formation; GS4: flowering; GS5: fruiting.
