# Supplementary material for: Community Succession and Diversity Variation of Endophytic and Rhizosphere Soil Bacteria Across Gastrodia elata Seed Formation Stages
Source: Biology (Basel). 2026 May 25;15(11):829. doi: 10.3390/biology15110829 (PMC13255848; doi:10.3390/biology15110829)
Supplement: Supplementary file 1 [file biology-15-00829-s001.zip › Figure S4. Community analysis pielot of endophytic bacteria from GE at different developmental stages at genus level ..pdf]

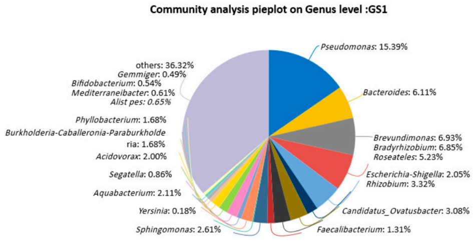

(A)

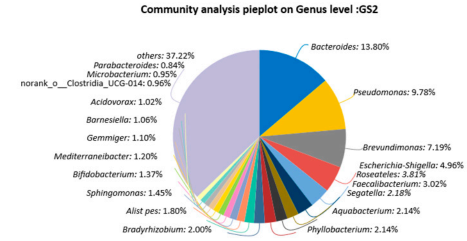

(B)

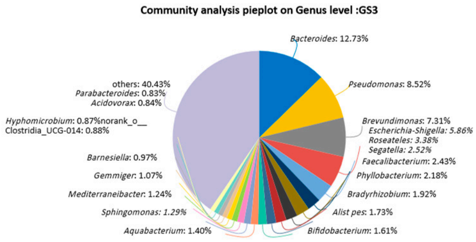

(C)

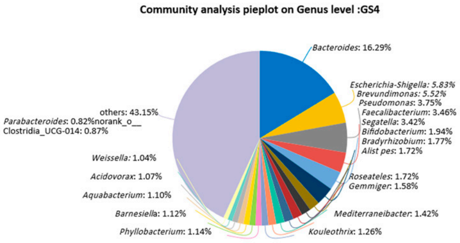

(D)

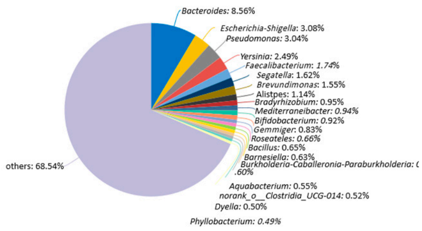

(E)

Figure S4. Community analysis pielot of endophytic bacteria from *GE* at different developmental stages at genus level . GS1: initial planting; GS2: seedling emergence; GS3: bud formation; GS4: flowering; GS5: fruiting. Different colors represent different species, and the area of each pie segment indicates the percentage proportion of the corresponding genus.
