# Supplementary material for: Community Succession and Diversity Variation of Endophytic and Rhizosphere Soil Bacteria Across Gastrodia elata Seed Formation Stages
Source: Biology (Basel). 2026 May 25;15(11):829. doi: 10.3390/biology15110829 (PMC13255848; doi:10.3390/biology15110829)
Supplement: Supplementary file 1 [file biology-15-00829-s001.zip › Figure S5. Stacked bar plot of endophytic bacterial relative abundance at the Phylum level in different tissues across five seed developmental stages (GS1–GS5) of GEpdf.pdf]

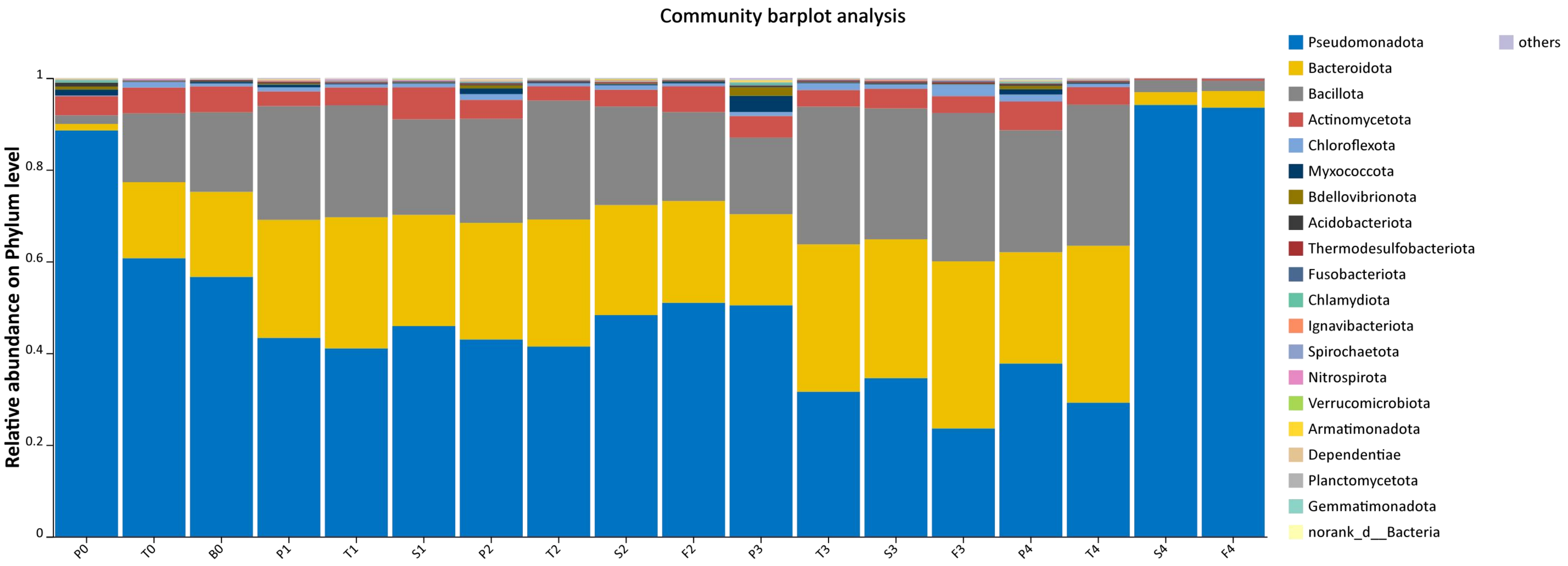

Figure S5. Stacked bar plot of endophytic bacterial relative abundance at the Phylum level in different tissues across five seed developmental stages (GS1–GS5) of *GE*. Tissue codes: epidermis (P0–P4), internal tissue (T0–T4), and stem (S0–S4) correspond to GS1–GS5, respectively; reproductive tissues (F2–F4: floral bud stalk, flower, seed) correspond to GS3–GS5, respectively.
