# Supplementary material for: Community Succession and Diversity Variation of Endophytic and Rhizosphere Soil Bacteria Across Gastrodia elata Seed Formation Stages
Source: Biology (Basel). 2026 May 25;15(11):829. doi: 10.3390/biology15110829 (PMC13255848; doi:10.3390/biology15110829)
Supplement: Supplementary file 1 [file biology-15-00829-s001.zip › Figure S6. Community analysis pieplot of endophytic bacteria in differend tissues at different stages of GE at phylum level.pdf]

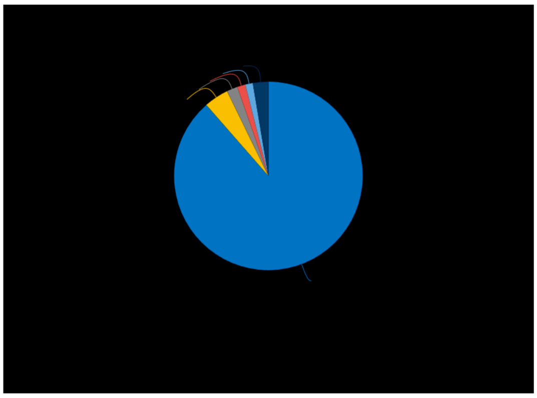

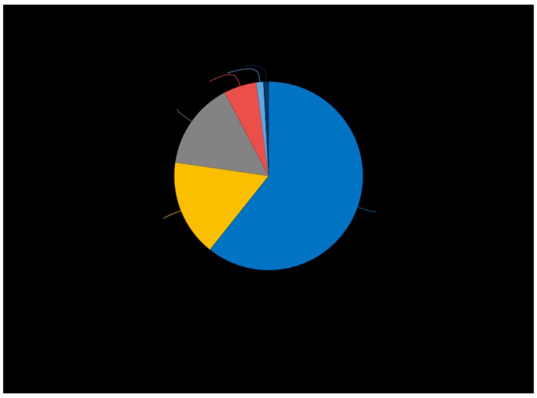

(A) (B)

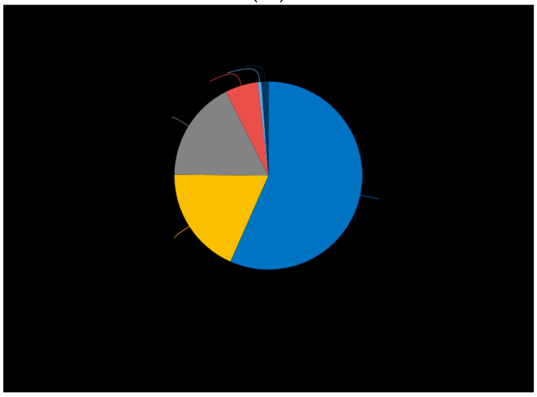

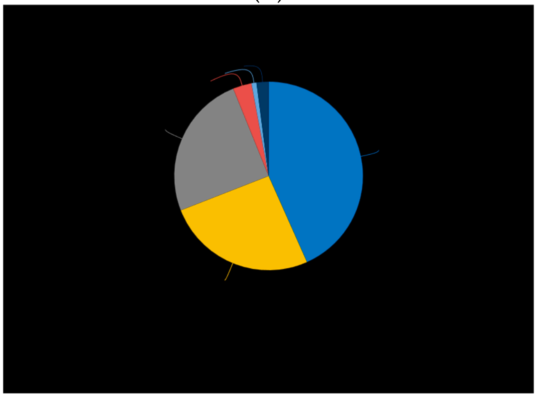

(C) (D)

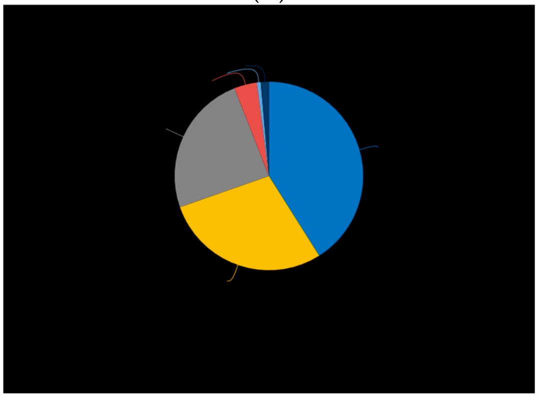

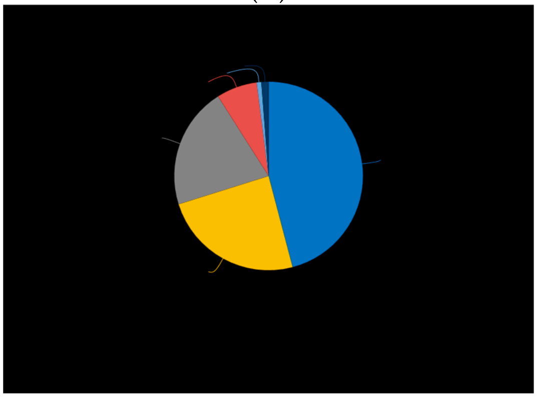

(E) (F)

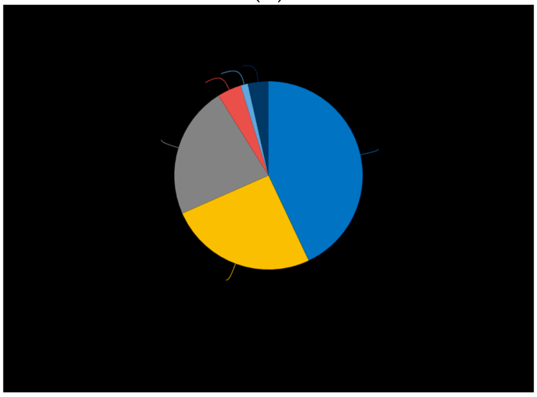

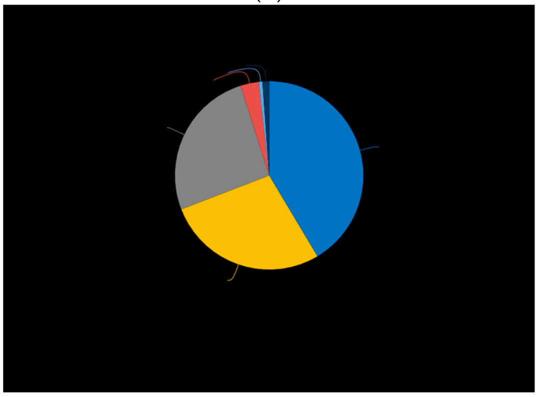

(G) (H)

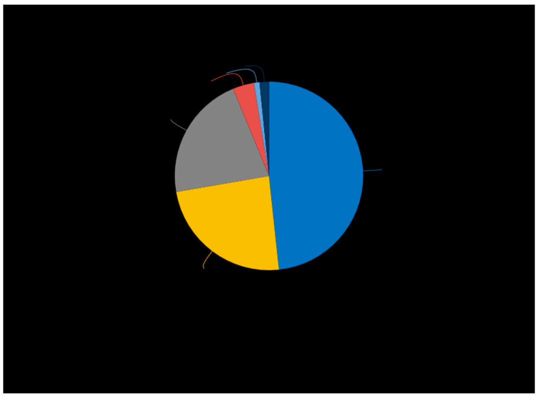

(I)

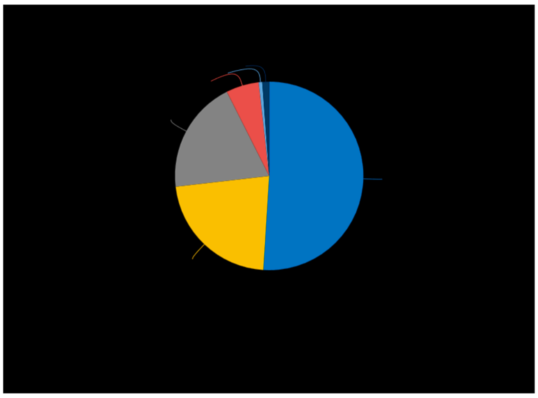

(J)

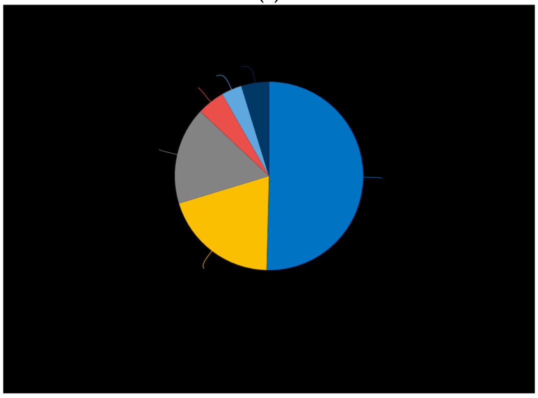

(K)

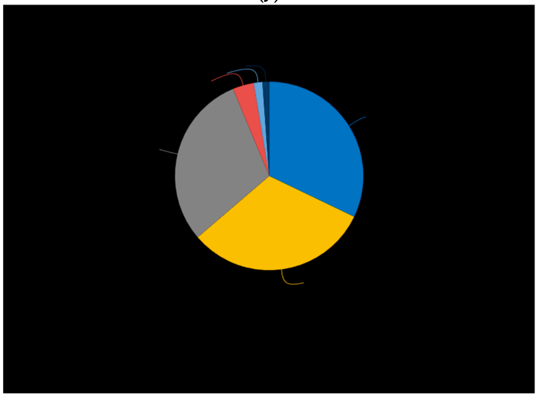

(L)

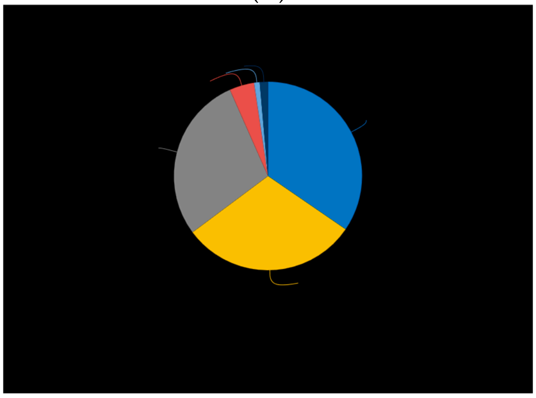

(M)

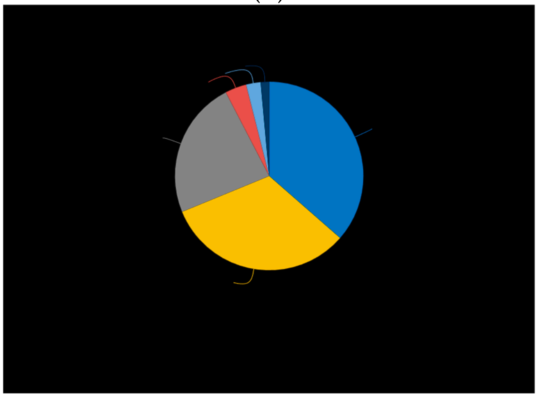

(N)

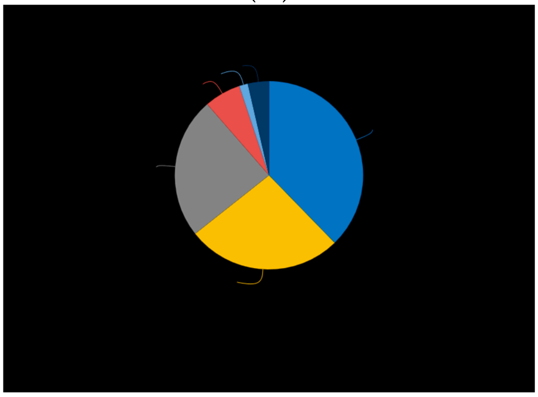

(O)

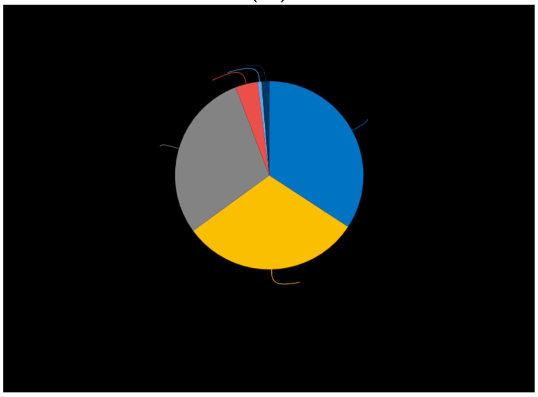

(P)

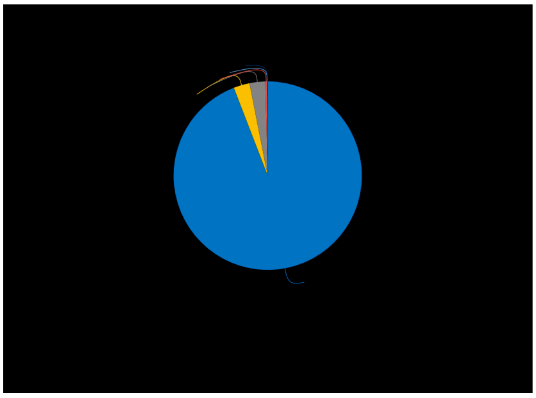

(Q)

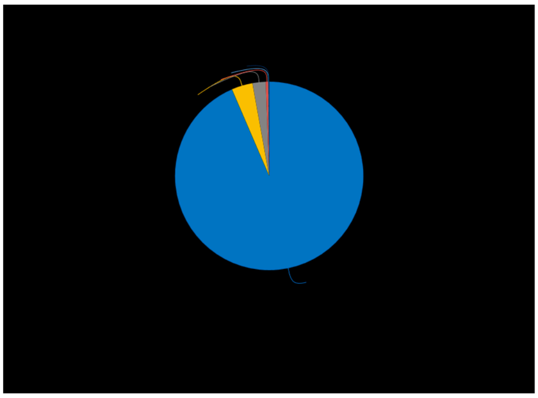

(R)

Figure S6. Community analysis pieplot of endophytic bacteria in differend tissues at different stages of *GE* at phylum level. Tissue codes: epidermis (P0 – P4), internal tissue (T0 – T4), and stem (S0 – S4) correspond to GS1 – GS5, respectively; reproductive tissues (F2 – F4: floral bud stalk, flower, seed) correspond to GS3 – GS5, respectively. Different colors represent different species, and the area of each pie segment indicates the percentage proportion of the corresponding phylum.

.

.
