# Supplementary material for: Community Succession and Diversity Variation of Endophytic and Rhizosphere Soil Bacteria Across Gastrodia elata Seed Formation Stages
Source: Biology (Basel). 2026 May 25;15(11):829. doi: 10.3390/biology15110829 (PMC13255848; doi:10.3390/biology15110829)
Supplement: Supplementary file 1 [file biology-15-00829-s001.zip › Figure S7. Community analysis pielot of endophytic bacteria endophytic bacteria at the genus level in differend tissues at different stages of GE.pdf]

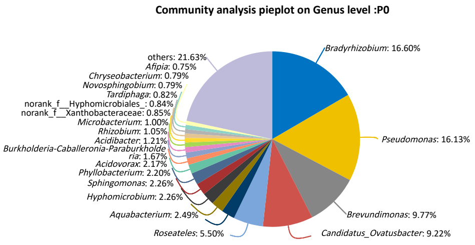
Community analysis pieplot on Genus level :P0
Bradyrhizobium: 16.60%
Pseudomonas: 16.13%
Brevundimonas: 9.77%
Candidatus_Ovatusbacter: 9.22%
Roseateles: 5.50%
Aquabacterium: 2.49%
Hyphomicrobium: 2.26%
Sphingomonas: 2.26%
Phyllobacterium: 2.20%
Acidovorax: 2.17%
Burkholderia-Caballeronia-Paraburkholde
ria: 1.67%
Acidibacter: 1.21%
Rhizobium: 1.05%
Microbacterium: 1.00%
norank_f__Xanthobacteraceae: 0.85%
norank_f__Hyphomicrobiales_: 0.84%
Tardiphaga: 0.82%
Novosphingobium: 0.79%
Chryseobacterium: 0.79%
Afipia: 0.75%
others: 21.63%

(A)

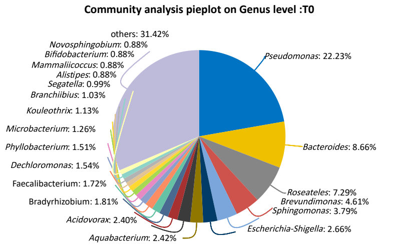
Community analysis pieplot on Genus level :T0
others: 31.42%
Novosphingobium: 0.88%
Bifidobacterium: 0.88%
Mammaliicoccus: 0.88%
Alistipes: 0.88%
Segatella: 0.99%
Branchiibius: 1.03%
Kouleothrix: 1.13%
Microbacterium: 1.26%
Phyllobacterium: 1.51%
Dechloromonas: 1.54%
Faecalibacterium: 1.72%
Bradyrhizobium: 1.81%
Acidovorax: 2.40%
Aquabacterium: 2.42%
Pseudomonas: 22.23%
Bacteroides: 8.66%
Roseateles: 7.29%
Brevundimonas: 4.61%
Sphingomonas: 3.79%
Escherichia-Shigella: 2.66%

(B)

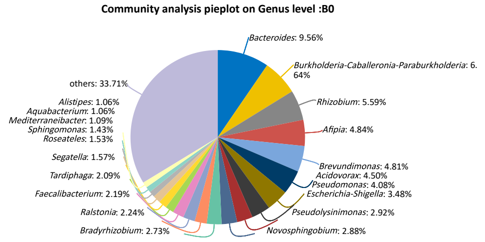
Community analysis pieplot on Genus level :B0
Bacteroides: 9.56%
Burkholderia-Caballeronia-Paraburkholderia: 6.
64%
Rhizobium: 5.59%
Afipia: 4.84%
Brevundimonas: 4.81%
Acidovorax: 4.50%
Pseudomonas: 4.08%
Escherichia-Shigella: 3.48%
Pseudolysinimonas: 2.92%
Novosphingobium: 2.88%
Bradyrhizobium: 2.73%
Ralstonia: 2.24%
Faecalibacterium: 2.19%
Tardiphaga: 2.09%
Segatella: 1.57%
Roseateles: 1.53%
Sphingomonas: 1.43%
Mediterraneibacter: 1.09%
Aquabacterium: 1.06%
Alistipes: 1.06%
others: 33.71%

(C)

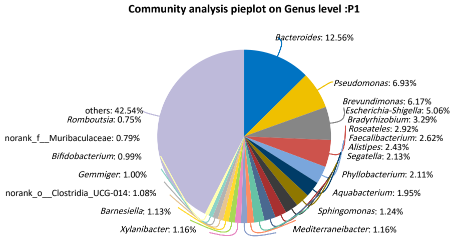
Community analysis pieplot on Genus level :P1
Bacteroides: 12.56%
Pseudomonas: 6.93%
Brevundimonas: 6.17%
Escherichia-Shigella: 5.06%
Bradyrhizobium: 3.29%
Roseateles: 2.92%
Faecalibacterium: 2.62%
Alistipes: 2.43%
Segatella: 2.13%
Phyllobacterium: 2.11%
Aquabacterium: 1.95%
Sphingomonas: 1.24%
Mediterraneibacter: 1.16%
Xylanibacter: 1.16%
Barnesiella: 1.13%
norank_o__Clostridia_UCG-014: 1.08%
Gemmiger: 1.00%
Bifidobacterium: 0.99%
norank_f__Muribaculaceae: 0.79%
Romboutsia: 0.75%
others: 42.54%

(D)

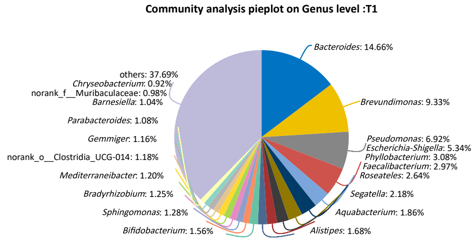
Community analysis pieplot on Genus level :T1
Bacteroides: 14.66%
Brevundimonas: 9.33%
Pseudomonas: 6.92%
Escherichia-Shigella: 5.34%
Phyllobacterium: 3.08%
Faecalibacterium: 2.97%
Roseateles: 2.64%
Segatella: 2.18%
Aquabacterium: 1.86%
Alistipes: 1.68%
Bifidobacterium: 1.56%
Sphingomonas: 1.28%
Bradyrhizobium: 1.25%
Mediterraneibacter: 1.20%
norank_o__Clostridia_UCG-014: 1.18%
Gemmiger: 1.16%
Parabacteroides: 1.08%
Barnesiella: 1.04%
norank_f__Muribaculaceae: 0.98%
Chryseobacterium: 0.92%
others: 37.69%

(E)

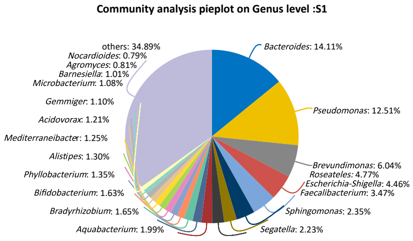
Community analysis pieplot on Genus level :S1
others: 34.89%
Nocardioides: 0.79%
Agromyces: 0.81%
Barnesiella: 1.01%
Microbacterium: 1.08%
Gemmiger: 1.10%
Acidovorax: 1.21%
Mediterraneibacter: 1.25%
Alistipes: 1.30%
Phyllobacterium: 1.35%
Bifidobacterium: 1.63%
Bradyrhizobium: 1.65%
Aquabacterium: 1.99%
Bacteroides: 14.11%
Pseudomonas: 12.51%
Brevundimonas: 6.04%
Roseateles: 4.77%
Escherichia-Shigella: 4.46%
Faecalibacterium: 3.47%
Sphingomonas: 2.35%
Segatella: 2.23%

(F)

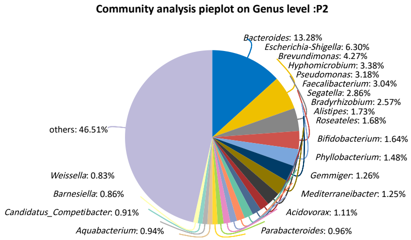
Community analysis pieplot on Genus level :P2
Bacteroides: 13.28%
Escherichia-Shigella: 6.30%
Brevundimonas: 4.27%
Hyphomicrobium: 3.38%
Pseudomonas: 3.18%
Faecalibacterium: 3.04%
Segatella: 2.86%
Bradyrhizobium: 2.57%
Alistipes: 1.73%
Roseateles: 1.68%
Bifidobacterium: 1.64%
Phyllobacterium: 1.48%
Gemmiger: 1.26%
Mediterraneibacter: 1.25%
Acidovorax: 1.11%
Parabacteroides: 0.96%
Aquabacterium: 0.94%
Candidatus_Competibacter: 0.91%
Barnesiella: 0.86%
Weissella: 0.83%
others: 46.51%

(G)

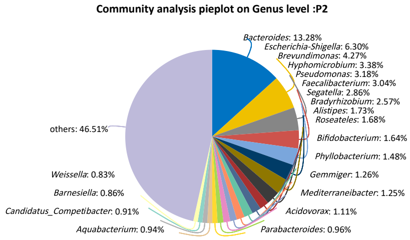
Community analysis pieplot on Genus level :P2
Bacteroides: 13.28%
Escherichia-Shigella: 6.30%
Brevundimonas: 4.27%
Hyphomicrobium: 3.38%
Pseudomonas: 3.18%
Faecalibacterium: 3.04%
Segatella: 2.86%
Bradyrhizobium: 2.57%
Alistipes: 1.73%
Roseateles: 1.68%
Bifidobacterium: 1.64%
Phyllobacterium: 1.48%
Gemmiger: 1.26%
Mediterraneibacter: 1.25%
Acidovorax: 1.11%
Parabacteroides: 0.96%
Aquabacterium: 0.94%
Candidatus_Competibacter: 0.91%
Barnesiella: 0.86%
Weissella: 0.83%
others: 46.51%

(H)

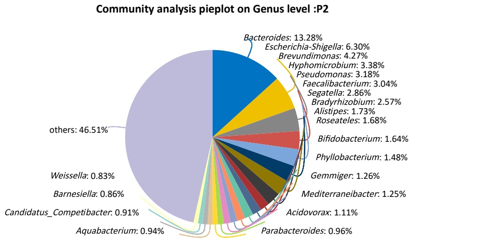
Community analysis pieplot on Genus level :P2
Bacteroides: 13.28%
Escherichia-Shigella: 6.30%
Brevundimonas: 4.27%
Hyphomicrobium: 3.38%
Pseudomonas: 3.18%
Faecalibacterium: 3.04%
Segatella: 2.86%
Bradyrhizobium: 2.57%
Alistipes: 1.73%
Roseateles: 1.68%
Bifidobacterium: 1.64%
Phyllobacterium: 1.48%
Gemmiger: 1.26%
Mediterraneibacter: 1.25%
Acidovorax: 1.11%
Parabacteroides: 0.96%
Aquabacterium: 0.94%
Candidatus_Competibacter: 0.91%
Barnesiella: 0.86%
Weissella: 0.83%
others: 46.51%

(I)

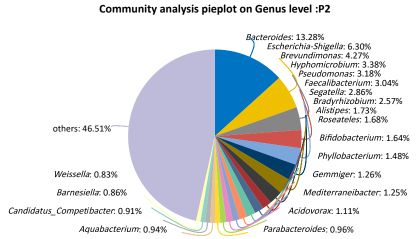
Community analysis pieplot on Genus level :P2
Bacteroides: 13.28%
Escherichia-Shigella: 6.30%
Brevundimonas: 4.27%
Hyphomicrobium: 3.38%
Pseudomonas: 3.18%
Faecalibacterium: 3.04%
Segatella: 2.86%
Bradyrhizobium: 2.57%
Alistipes: 1.73%
Roseateles: 1.68%
Bifidobacterium: 1.64%
Phyllobacterium: 1.48%
Gemmiger: 1.26%
Mediterraneibacter: 1.25%
Acidovorax: 1.11%
Parabacteroides: 0.96%
Aquabacterium: 0.94%
Candidatus_Competibacter: 0.91%
Barnesiella: 0.86%
Weissella: 0.83%
others: 46.51%

(J)

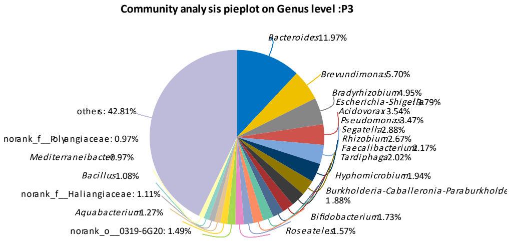
Community analysis pieplot on Genus level :P3
Bacteroides: 11.97%
Brevundimonas: 5.70%
Bradyrhizobium: 4.95%
Escherichia-Shigella: 3.79%
Acidovorax: 3.54%
Pseudomonas: 3.47%
Segatella: 2.88%
Rhizobium: 2.67%
Faecalibacterium: 2.17%
Tardiphaga: 2.02%
Hyphomicrobium: 1.94%
Burkholderia-Caballeronia-Paraburkholde
1.88%
Bifidobacterium: 1.73%
Roseateles: 1.57%
norank_o__0319-6G20: 1.49%
Aquabacterium: 1.27%
norank_f__Haliangiaceae: 1.11%
Bacillus: 1.08%
Mediterraneibacter: 0.97%
norank_f__Polyangiaceae: 0.97%
others: 42.81%

(K)

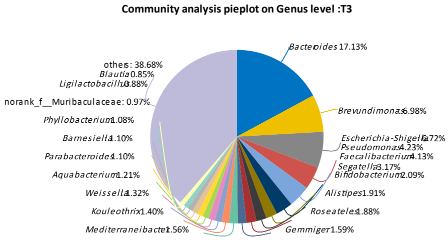
Community analysis pieplot on Genus level :T3
Bacteroides: 17.13%
Brevundimonas: 6.98%
Escherichia-Shigella: 6.72%
Pseudomonas: 4.23%
Faecalibacterium: 4.13%
Segatella: 3.17%
Bifidobacterium: 2.09%
Alistipes: 1.91%
Roseateles: 1.88%
Gemmiger: 1.59%
Mediterraneibacter: 1.56%
Kouleothrix: 1.40%
Weissella: 1.32%
Aquabacterium: 1.21%
Parabacteroides: 1.10%
Barnesiella: 1.10%
Phyllobacterium: 1.08%
norank_f__Muribaculaceae: 0.97%
Ligilactobacillus: 0.88%
Blautia: 0.85%
others: 38.68%

(L)

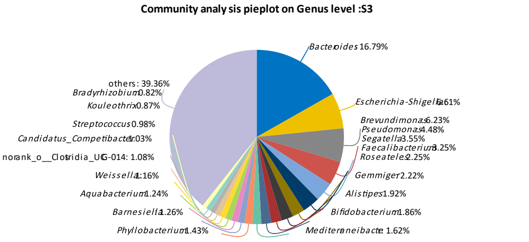
Community analysis pieplot on Genus level :S3
Bacteroides: 16.79%
Escherichia-Shigella: 6.61%
Brevundimonas: 6.23%
Pseudomonas: 4.48%
Segatella: 3.55%
Faecalibacterium: 3.25%
Roseateles: 2.25%
Gemmiger: 2.22%
Alistipes: 1.92%
Bifidobacterium: 1.86%
Mediterraneibacter: 1.62%
Phyllobacterium: 1.43%
Barnesiella: 1.26%
Aquabacterium: 1.24%
Weissella: 1.16%
norank_o__Clostridia_UCG-014: 1.08%
Candidatus_Competibacter: 1.03%
Streptococcus: 0.98%
Kouleothrix: 0.87%
Bradyrhizobium: 0.82%
others: 39.36%

(M)

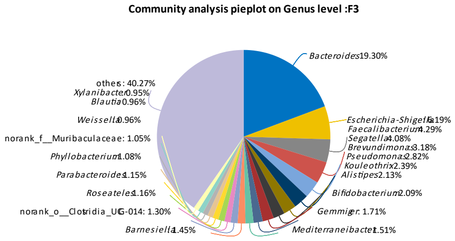
Community analysis pieplot on Genus level :F3
Bacteroides: 19.30%
Escherichia-Shigella: 6.19%
Faecalibacterium: 4.29%
Segatella: 4.08%
Brevundimonas: 3.18%
Pseudomonas: 2.82%
Kouleothrix: 2.39%
Alistipes: 2.13%
Bifidobacterium: 2.09%
Gemmiger: 1.71%
Mediterraneibacter: 1.51%
Barnesiella: 1.45%
norank_o__Clostridia_UCG-014: 1.30%
Roseateles: 1.16%
Parabacteroides: 1.15%
Phyllobacterium: 1.08%
norank_f__Muribaculaceae: 1.05%
Weissella: 0.96%
Blautia: 0.96%
Xylanibacter: 0.95%
others: 40.27%

(N)

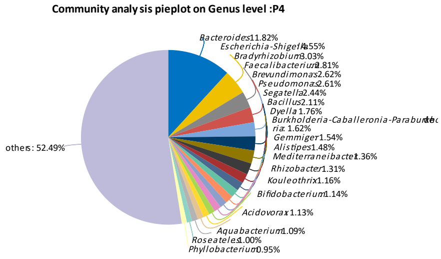
Community analysis pieplot on Genus level :P4
Bacteroides: 11.82%
Escherichia-Shigella: 4.55%
Bradyrhizobium: 3.03%
Faecalibacterium: 2.81%
Brevundimonas: 2.62%
Pseudomonas: 2.61%
Segatella: 2.44%
Bacillus: 2.11%
Dyella: 1.76%
Burkholderia-Caballeronia-Paraburkholde
ria: 1.62%
Gemmiger: 1.54%
Alistipes: 1.48%
Mediterraneibacter: 1.36%
Rhizobacter: 1.31%
Kouleothrix: 1.16%
Bifidobacterium: 1.14%
Acidovorax: 1.13%
Aquabacterium: 1.09%
Roseateles: 1.00%
Phyllobacterium: 0.95%
others: 52.49%

(O)

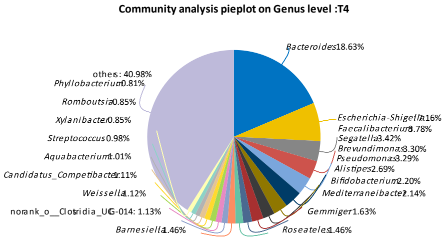
Community analysis pieplot on Genus level :T4
Bacteroides: 18.63%
Escherichia-Shigella: 7.16%
Faecalibacterium: 3.78%
Segatella: 3.42%
Brevundimonas: 3.30%
Pseudomonas: 3.29%
Alistipes: 2.69%
Bifidobacterium: 2.20%
Mediterraneibacter: 2.14%
Gemmiger: 1.63%
Roseateles: 1.46%
Barnesiella: 1.46%
norank_o__Clostridia_UCG-014: 1.13%
Weissella: 1.12%
Candidatus_Competibacter: 1.11%
Aquabacterium: 1.01%
Streptococcus: 0.98%
Xylanibacter: 0.85%
Romboutsia: 0.85%
Phyllobacterium: 0.81%
others: 40.98%

(P)

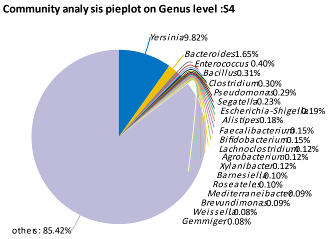

(Q)

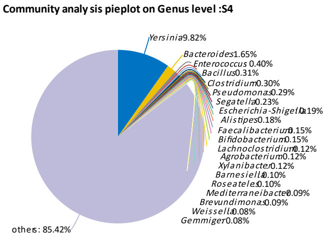

(R)

Figure S7. Community analysis pielot of endophytic bacteria endophytic bacteria at the genus level in differend tissues at different stages of *GE*. Tissue codes: epidermis (P0–P4), internal tissue (T0–T4), and stem (S0–S4) correspond to GS1–GS5, respectively; reproductive tissues (F2–F4: floral bud stalk, flower, seed) correspond to GS3–GS5, respectively.Different colors represent different species, and the area of each pie segment indicates the percentage proportion of the corresponding genus.
