# Supplementary material for: Community Succession and Diversity Variation of Endophytic and Rhizosphere Soil Bacteria Across Gastrodia elata Seed Formation Stages
Source: Biology (Basel). 2026 May 25;15(11):829. doi: 10.3390/biology15110829 (PMC13255848; doi:10.3390/biology15110829)
Supplement: Supplementary file 1 [file biology-15-00829-s001.zip › Figure S8.Phylum‐level taxonomic composition of rhizosphere soil bacterial communities across five seed developmental stages of GE .pdf]

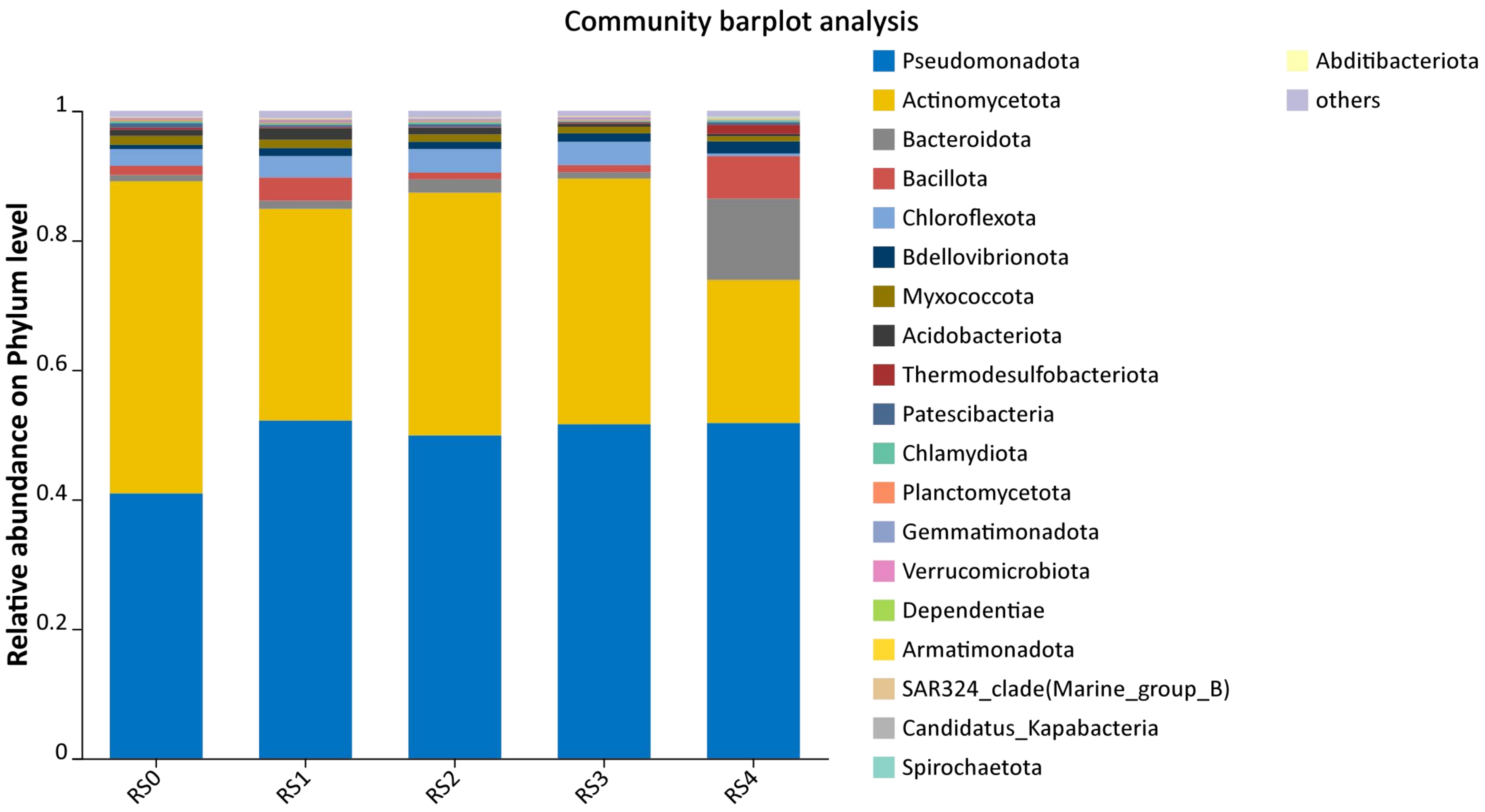


Figure S8.Phylum - level taxonomic composition of rhizosphere soil bacterial communities across five seed developmental stages of *GE* (RS0–RS4: initial planting, seedling emergence, bud formation, flowering, and fruiting).
