# Supplementary material for: Community Succession and Diversity Variation of Endophytic and Rhizosphere Soil Bacteria Across Gastrodia elata Seed Formation Stages
Source: Biology (Basel). 2026 May 25;15(11):829. doi: 10.3390/biology15110829 (PMC13255848; doi:10.3390/biology15110829)
Supplement: Supplementary file 1 [file biology-15-00829-s001.zip › Figure S9. Community analysis pielot of rhizospheric soil bacteria from GE at different developmental stages at phylum level.pdf]

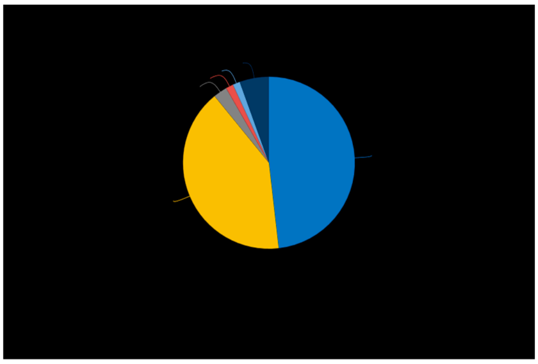

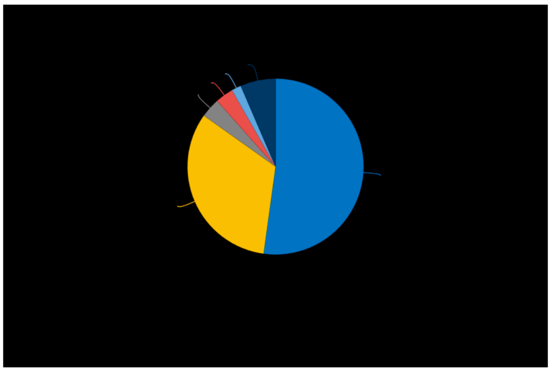

(A) (B)

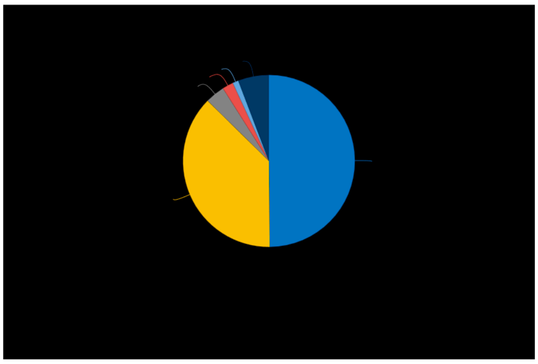

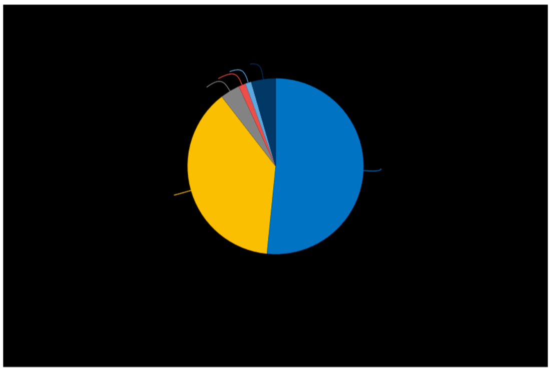

(C) (D)

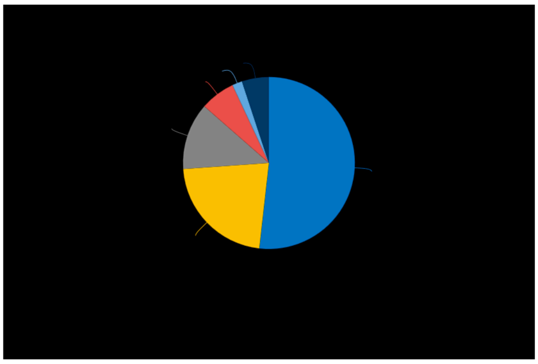

(E)

Figure S9. Community analysis pielot of rhizospheric soil bacteria from *GE* at different developmental stages at phylum level (RS0–RS4: initial planting, seedling emergence, bud formation, flowering, and fruiting).Different colors represent different species, and the area of each pie segment indicates the percentage proportion of the corresponding phylum.
