# Supplementary material for: Community Succession and Diversity Variation of Endophytic and Rhizosphere Soil Bacteria Across Gastrodia elata Seed Formation Stages
Source: Biology (Basel). 2026 May 25;15(11):829. doi: 10.3390/biology15110829 (PMC13255848; doi:10.3390/biology15110829)
Supplement: Supplementary file 1 [file biology-15-00829-s001.zip › Table S1. Sample grouping and coding scheme for G. elata across five seed formation developmental stages.pdf]

Table S1. Sample grouping and coding scheme for G. elata across five seed formation developmental stages.

Growth stage	Group coding	Collect samples	Sample coding
Initial planting	Growth stage1 (GS1)	Epidermis 0	P0
		Internal tissues 0	T0
		Floral bud stalk 0	B0
		Rhizosphere soil 0	RS0
Seeding emergence	Growth stage2 (GS2)	Epidermis 1	P1
		Internal tissues 1	T1
		Stem 1	S1
		Rhizosphere soil 1	RS1
Bud formation	Growth stage 3 (GS3)	Epidermis 2	P2
		Internal tissues 2	T2
		Stem 2	S2
		Floral bud 2	F2
		Rhizosphere soil 2	RS2
Flowering	Growth stage4 (GS4)	Epidermis 3	P3
		Internal tissues 3	T3
		Stem 3	S3
		Flower 3	F3
		Rhizosphere soil 3	RS3
Fruiting	Growth stage 5 (GS5)	Epidermis 4	P4
		Internal tissues 4	T4
		Stem 4	S4
		Seed 4	F4
		Rhizosphere soil 4	RS4
