# Supplementary material for: Community Succession and Diversity Variation of Endophytic and Rhizosphere Soil Bacteria Across Gastrodia elata Seed Formation Stages
Source: Biology (Basel). 2026 May 25;15(11):829. doi: 10.3390/biology15110829 (PMC13255848; doi:10.3390/biology15110829)
Supplement: Supplementary file 1 [file Figure S10. Community analysis pielot of rhizospheric soil bacteria from GE at different developmental stages at genus level.pdf › Figure S10. Community analysis pielot of rhizospheric soil bacteria from GE at different developmental stages at genus level.pdf]

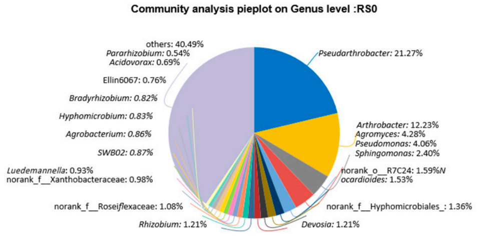

(A)

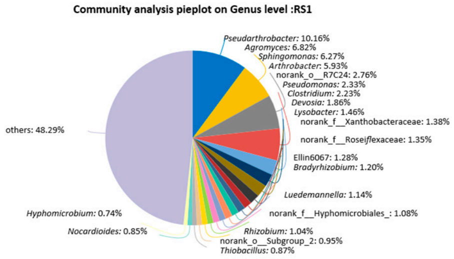

(B)

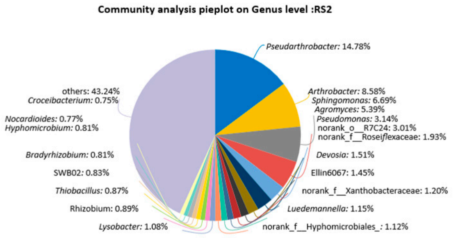

(C)

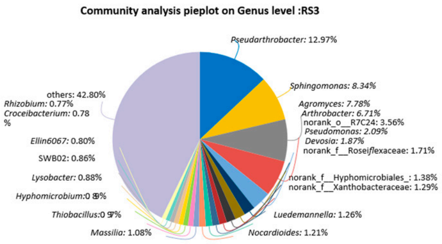

(D)

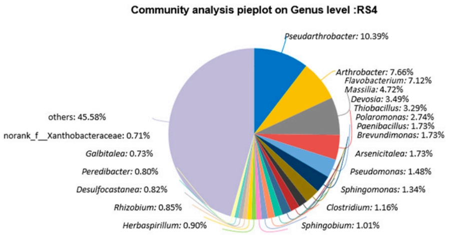

(E)

Figure S10. Community analysis pielot of rhizospheric soil bacteria from *GE* at different developmental stages at genus level (RS0–RS4: initial planting, seedling emergence, bud formation, flowering, and fruiting). Different colors represent different species, and the area of each pie segment indicates the percentage proportion of the corresponding genus.
